# Molecular characterization of human group A rotavirus genotypes circulating in Rawalpindi, Islamabad, Pakistan during 2015-2016

**DOI:** 10.1371/journal.pone.0220387

**Published:** 2019-07-30

**Authors:** Asma Sadiq, Nazish Bostan, Habib Bokhari, Jelle Matthijnssens, Kwe Claude Yinda, Saqlain Raza, Tayyab Nawaz

**Affiliations:** 1 Department of Biosciences, COMSATS University (CUI), Tarlai Kalan, Chak Shahzad, Islamabad, Pakistan; 2 KU Leuven-University of Leuven, Department of Microbiology and Immunology, Rega Institute for Medical Research, Laboratory of Viral Metagenomics, Leuven, Belgium; Defense Threat Reduction Agency, UNITED STATES

## Abstract

Group A rotaviruses (RVA) are one of the major causes of acute gastroenteritis (AGE) in young children worldwide. Owing to lack of proper surveillance programs and health facilities, developing countries of Asia and Africa carry a disproportionately heavy share of the RVA disease burden. The aim of this hospital-based study was to investigate the circulation of RVA genotypes in Rawalpindi and Islamabad, Pakistan in 2015 and 2016, prior to the implementation of RVA vaccine. 639 faecal samples collected from children under 10 years of age hospitalized with AGE were tested for RVA antigen by ELISA. Among 171 ELISA positive samples, 143 were successfully screened for RT-PCR and sequencing. The prevalence of RVA was found to be 26.8% with the highest frequency (34.9%) found among children of age group 6–11 months. The most predominant circulating genotypes were G3P[8] (22.4%) followed by G12P[6] (20.3%), G2P[4] (12.6%), G1P[8] (11.9%), G9P[6] (11.9%), G3P[4] (9.1%), G1P[6] (4.2%), G9P[8] (4.2%), and G3P[6] (0.7%). A single mixed genotype G1G3P[8] was also detected. The findings of this study provide baseline data, that will help to assess if future vaccination campaigns using currently available RVA vaccine will reduce RVA disease burden and instigate evolutionary changes in the overall RVA biology. The high prevalence of RVA infections in Pakistan require to improve and strengthen the surveillance and monitoring system for RVA. This will provide useful information for health authorities in planning public health care strategies to mitigate the disease burden caused by RVA.

## Introduction

Group A rotaviruses (RVAs) belong to family *Reoviridae* are the leading cause of fatal dehydrating diarrhea in infants and young children worldwide particularly in the developing countries of Africa and Asia [1–4]. The rotavirus is a 11 segmented double-stranded RNA (dsRNA) virus encoding six structural proteins (VP1-VP4, VP6, VP7) and six non-structural proteins (NSP1-NSP6) [5]. The segmented nature of its genome allows RVs to adopt remarkable genetic diversity [6]. Based on the sequence of VP7 and VP4 genes, RVAs are classified into G and P-genotypes, respectively. Currently, 36 G and 51 P-types have been reported worldwide [7]. The most common G/P genotype combinations causing 90% infections in in humans are G1P[8], G2P[4], G3P[8], G4P[8], G9P[8] and G12P[8] [8,9]. Vaccination is an effective measure to combat RVA infections. Two live oral rotavirus vaccines (Rotarix, GlaxoSmithKline and RotaTeq, Merck & Co) have been licensed and are available in the market since 2006 [10,11]. Rotarix is a monovalent oral live-attenuated vaccine and RotaTeq is a pentavalent human-bovine reassortant vaccine [12].

Globally, acute gastroenteritis caused by RVA is a major public health concern, estimated to cause 215,000 deaths in children of <5 years of age in 2013 [13,14]. Four countries (India, Nigeria, Pakistan and Democratic Republic of the Congo) accounted for almost half (45%) of deaths due to RVA in 2013 [14,15]. In the last decade, a large research data obtained on RVA associated disease burden and epidemiology helped in the implementations of more effective health policies including RVA vaccination [16]. In 2009, WHO has recommended the inclusion of RVA vaccines (Rotarix and RotaTeq) in the Extended program on immunization (EPI) worldwide [17,18]. As of August 2018, 98 countries have introduced rotavirus vaccine in their national immunization program including six high disease burden countries (Afghanistan, Angola, Ethiopia, India, Kenya and Pakistan) [19]. In the developed countries of Europe and America, both vaccines resulted in a significant drop in diarrhea associated deaths but proved to be less effective in developing countries of Asia and Africa [16,20]. The proposed hypothesis for lower vaccine efficacy in low income countries include interaction with maternal antibodies, malnutrition, host concomitant infections, RVA genotypes diversity and difference in gut micro flora [21,22].

In Pakistan, the under-five child mortality rate is 67.6 deaths per 100000 children due to RVA diarrhea [13]. The data on the molecular epidemiology and genotype diversity of RVA in Pakistan is limited [23]. The previous studies have reported the RVA detection rate of 29–63% with G1P[8], G2P[4] and G9P[8] as the highly prevalent genotypes circulating in Pakistan during 2009–2013 [23–30]. In 2014 the proportion of G3 increased dramatically, and G3P[8] became the dominant strain [31]. To reduce the childhood mortality, Pakistan began a phase RVA vaccine introduction in early 2017 in Punjab province [32]. In 2018 with the support of GAVI, the Vaccine Alliance, the government of Pakistan has included RVA vaccination in the EPI schedule of each of the four provinces (Punjab, Khyber-Pakhtunkhwa, Sindh and Baluchistan) [33–37]. This means, the children who were previously excluded from the benefits of rotavirus vaccines are now included for vaccination.

Our study aimed to investigate the prevalence and diversity of RVA strains in two major metropolitan cities (Rawalpindi and Islamabad) of Pakistan in 2015 and 2016. The results of this pre-vaccination study will provide reasonable data set for the researchers and public health authorities for comparative analysis with the post-vaccination studies in the future. That will eventually help to assess the efficacy of vaccination and the potential effect on the strain diversity and evolution due to vaccine selective pressure in Pakistan.

## Materials and methods

### Study sites

The following two hospitals have been selected to be the sites for the current study. Pakistan Institute of Medical Sciences (PIMS) is the major tertiary care hospital of the capital city Islamabad with 9000 cases in outpatient department (OPD) per day and more than 200 hospitalizations each day. The hospital provides health care to more than 10 million individuals from urban, peri-urban and rural settings of the country, and is a referral hospital for patients from all over Pakistan. Similarly, another tertiary care setup in Rawalpindi is the General Hospital (now called Benazir Bhutto Hospital (BBH). It has up to 2500 patient visits in OPDs per day. It is a public-sector hospital with a dedicated paediatric unit including a diarrhea ward. These are major metropolitan cities in the country with a large population size (4.5 million). Hence, the results obtained will probably reflect a large section of the country’s population.

### Ethical approval and consent

The sampling was carried out after gaining informed consents from the parents/guardians of the study participants. Ethical approval was obtained from the ethical committee of PIMS, Benazir Bhutto Shaheed Hospital (BBH) and Internal Review Board (IRB) of COMSATS University, Islamabad, Pakistan.

### Sample collection

A total of 639 stool samples were collected from January 2015 to December 2016 from children less than 10 years of age, hospitalized/visited or received IV rehydration treatment in the emergency paediatric ward of two hospitals, BBH in Rawalpindi and PIMS in Islamabad.

Samples were collected in stool collection vials from patients with three or more watery non-bloody stools in a 24 hours period excluding non-infectious bloody diarrheal cases in accordance with WHO case standard guideline [38]. All the samples were initially kept at in the hospital at -20˚C and transported under cold chain to the microbiology and public health laboratory, COMSATS University, Islamabad. Upon arrival at COMSATS, all stool samples were stored at −80˚C, until serological and molecular analysis. Demographic and clinical data including age, gender, residence, hospital admission date, diarrhea onset date, date of stool sample collection, vomiting (duration and episodes per day), diarrhea (duration and episodes per day) and body temperature of the patients was recorded.

### Detection of RVA by enzyme immunoassay (ELISA)

Faecal suspensions (10–20%) were prepared by adding 100–200 mg of faecal sample in 1 ml of universal transport medium (UTM, Copan Diagnostics) in a clean 1.5 ml centrifuge tube. The individual faecal suspensions were tested for the detection of RVA antigen using the ProSpectT Rotavirus ELISA Kit (Oxoid Ltd, UK). Stools positive for RVA were shipped to Rega Institute for Medical Research, Leuven, Belgium for further characterization. Specimens were packed in transport box with (ice packs) to maintain a temperature of 2–8 C˚.

The ELISA negative faecal samples were not under the scope of our study so, they were not further tested.

### RNA extraction

Viral RNA was extracted from 10% faecal suspension by using the QIAamp Viral RNA Mini Kit (Qiagen/Westburg, The Nederlands) according to manufacturer’s instructions. A negative extraction control with PBS and RVA positive control was included in the extraction procedure in each batch and quality of extracted RNA was checked through spectrophotometer.

### RT-PCR for VP7 and VP4 genes

The extracted RNA template was denatured for 2 minutes at 95°C followed by reverse transcriptase PCR (RT-PCR) using the One-step RT-PCR Kit (Qiagen/Westburg, The Nederlands). RT-PCR was carried out for both VP7 and VP4 gene fragments using consensus primers Beg9 and End9 [39] for VP7 and VP4_1-17F and Con2Deg for VP4 [40]. Samples negative for PCR using Beg9/End9 and VP4_1-17F/Con2Deg primer sets were further characterized by RT-PCR using the 2nd primer sets (VP7F and VP7R for VP7;VP4F and VP4R for VP4 [41,42], as there might be mutation in primer binding sites which can be captured by other set of primers. The primer sequences are given in the Table 1. The RT-PCR was carried out with an initial reverse transcription step of 30 min at 50°C followed by polymerase activation at 95°C for 15 min, 40 cycles of amplification (denaturation: 45s at 94°C; annealing: 45s at 45°C for VP4 and 45s at 50°C for VP7; product extension for 1 min at 72°C), followed by a final extension of 10 minutes at 72°C in a Biometra T3000 thermocycler (Biometra, Westburg). PCR products were run on a polyacrylamide gel along with a 50-bp DNA ladder (Sigma Aldrich), stained with EtBr (Sigma Aldrich) and visualized under UV-light. The sample preparation, amplification and end point analysis were kept physically separated. All steps of experiment i.e Extraction, PCR were done in a bio-safety cabinets. All the PCR runs included positive control, negative control and non-target control (reagent blank) to avoid false positive results. A rotavirus positive sample (Genotype G1P[8] was used as a positive control. Quality control sample were run in each batch to check the instrumental stability (S1 and S2 Figs).

**Table 1:** List of consensus primers utilized in this study.

**VP4 Primers**	
**Primer Name**	**Sequence (5'-3')**
VP4F	5' TAT GCT CCA GTN AAT TGG 3'
VP4R	5' ATT GCA TTT CTT TCC ATA ATG 3'
VP4_1-17F	5' gGC TAt aaa atg gct tcg c 3'
con2Deg	5' ATT TCG GAC CAT TTA TAA CC 3'
**VP7 Primers**	
**Primer Name**	**Sequence (5'-3')**
Beg9	5' GGC TTT AAA AGA GAG AAT TTC CGT CTG G 3'
End9	5' GGT CAC ATC ATA CAA TTC TAA TCT AAG 3'
VP7-F	5' ATG TAT GGT ATT GAA TAT ACC AC3'
VP7-R	5' AAC TTG CCA CCATTT TTT CC3'

### Nucleotide sequencing

The PCR product was purified using the ExoSAP clean-up kit (Thermofisher Scientific, USA). The PCR amplicons were then sequenced using the BigDye Cycle Sequencing Kit (Applied Biosystems, USA). The sequencing was performed with the same forward primers as were used for the RT-PCR [43]. After the sequencing reaction, an ethanol precipitation was performed and the final product was loaded in ABI PRISM 3130 automated sequencer (Applied Biosystems, USA) [44].

### Determination of RVA genotypes for VP7 and VP4 genes

The chromatogram obtained were analysed by using Chromas 2.6.4 (Technelysium, Queensland, Australia). The sequences were manually corrected and compared with other sequences available in Genbank using BLASTn. Their genotype were then obtained using the RVA online classification tool RotaC 2.0v (http://rotac.regatools.be/) [45]. The nucleotide sequences were submitted to GenBank and the accession numbers can be found in S1 Table.

### Phylogenetic analysis

Multiple sequence alignments were performed using ClustalW in MEGA version 6.06 [46]. Phylogenetic trees were constructed by Maximum Likelihood method with kimura-2-parameter model in MEGA 6.06 [46]. The statistical reliability was checked with using 1000 bootstrap replicates. Nucleotide and amino acid distances were calculated using the P Distance Model.

### Nucleotide sequence accession numbers

The VP7 and VP4 nucleotide sequences were submitted to the GenBank with following accession numbers: **(G1)** MH062755-MH062758, MH191269-MH191282 and MH277407-MH277409, **(G2)** MH109860-MH109868, MH182472-MH182475 and MH277404-MH277406, **(G3)** MH109807-MH109826 and MH279569-MH279593, **(G9)** MH109847-MH109859, MH109869-109870 and MH277394-MH277403, **(G12)** MH109827-MH109844 and MH255697-MH255708, **P[4]** MH109741-MH109754 and MH236884-MH236897, **P[6]** MH109777-MH109806 and MH255676-MH255696, **P[8]** MH109755-MH109776 and MH255709-MH255740.

### Statistical analysis

Statistical analyses were performed with Stata version 13 (StataCorp. 2013) [47]. A chi-square test was performed to test the possible association between gender difference and RVA status. Student’s *t*-test was performed to ascertain the equality of means of RVA positive and negative for continuous variables; age, weight, height, temperature, vomiting (duration and episodes), diarrhea (duration and episodes) and dehydration. Statistical significance was defined as *p* < 0.05.

## Results

### RVA positivity and socio-demographic characteristics

RVA was detected in 171 samples via ELISA out of the collected 639 diarrheal samples from children less than ten years of age presenting with AGE with an overall positivity rate of 26.8% (25.4% in 2015 and 29.3% in 2016). The RVA prevalence detected in BBH, Rawalpindi was 27.4% (96/350) and in PIMS, Islamabad was 26.0% (75/289). RT-PCR conducted on all ELISA positive samples showed that 83.6% (143/171) of these samples were positive by RT-PCR. There was no statistically significant gender difference in the distribution of RVA genotypes (*p*>0.05) (Table 2). There was a statistically significant difference between mean age (in months) of RVA gastroenteritis cases as detected by ELISA (*p*<0.05). Whereas, no significant difference (*p*>0.05) was found between different age groups positive for RVA through ELISA (Table 2). However, the highest prevalence was observed in children of 6–11 months of age followed by children of 12–23 months of age, while the lowest number of positive cases were observed in children older than 60 months. There was no significant difference found between anthropomorphic and clinical features (weight, height, temperature, diarrhea duration and episodes, vomiting duration and episodes and dehydration) and RVA infections (Table 2).

**Table 2:** Comparison of demographic and clinical features of children with RVA positive and negative gastroenteritis by ELISA.

ELISA				
	RV(+)	RV(-)	*p*-value	Total
Demographic and Clinical Characteristics	(n = 171)	(n = 468)		639 (100%)
Gender				
Male	98 (27.1%)	264 (72.9%)	0.84	362 (56.7%)
Female	73 (26.4%)	204 (73.6%)		277 (43.3%)
Mean age (months)	12.18 ± 19.61	17.16 ± 27.09	0.03*	16±5
Group age (months)[0–5]	54 (23%)	179 (77%)	0.18	233(36.5%)
[6–11]	68 (34.9%)	127 (65.1%)		195(30.5%)
[12–23]	32 (33.7%)	63 (66.3%)		95(14.9%)
[24–59]	11 (15%)	62 (85%)		73 (11.4%)
[60 +]	6 (14%)	37 (86%)		43(6.7%)
	**Mean ± SD**			
Weight (in kg)	6.94 ± 3.83	7.51 ± 5.17	0.19	
Height (in cm)	69.95 ± 14.36	71.12±15.59	0.39	
Temp (in C°)	37.79 ± 0.34	37.73 ± 0.38	0.11	
Vomiting Duration (days)	2.60 ± 1.59	2.54 ± 1.77	0.68	
Vomiting Episode/24hours	5.95 ± 5.19	5.99 ± 5.26	0.94	
Diarrhea Duration (days)	2.70 ± 1.53	2.69 ± 1.73	0.89	
Diarrhea Episode/24 hours	19.36 ± 5.59	19.94 ± 6.08	0.27	
Dehydration	0.53 ± 0.50	0.52 ± 0.50	0.74	

*P*<0.05 was considered statistically significant

### Prevalence of RVA genotypes

From the 171 RVA antigen-positive samples, 143(83.6%) were confirmed positive by RT-PCR and the remaining 16.4% were RT-PCR negative. All 143 RT-PCR positive samples were successfully genotyped for VP7 and 140 samples were genotyped for VP4. The most common G type was G3 followed by G12, G9, G2 and G1. A single mixed G-type (G1G3) was detected in one sample (0.7%). The most common P-type was P[8] followed by P[6], P[4] and P[Non-typeable]. The most prevalent genotype combination was G3P[8] followed by G12P[6], G2P[4], G1P[8], G9P[6] and G3P[4], whereas the least prevalent genotypes were G9P[8], G3P[6], G1G3P[8], and G9P[NT] (Table 3).

**Table 3:** Prevalence of RVA G/P genotypes among 143 children hospitalized in local hospitals of Pakistan in 2015 and 2016.

	P Genotype				
G Genotype	P[4]	P[6]	P[8]	P[NT]	Total
**G1**	-	6 (4.1%)	17 (11.9%)	-	23 (16.1%)
**G2**	18 (12.6%)	-	-	-	18 (12.6%)
**G3**	13 (9.1%)	1 (0.7%)	32 (22.4%)		46 (32.2%)
**G9**	-	17 (11.9%)	6 (4.2%)	3 (2.1%)	26 (18.2%)
**G12**	-	29 (20.3%)			29 (20.3%)
**G Mix (G1G3)**	-	-	1 (0.7%)		1 (0.7%)
**Total**	31 (21.7%)	53 (37.1%	56 (39.2%)	3 (2.1%)	143 (100%)

**Fig 1:**
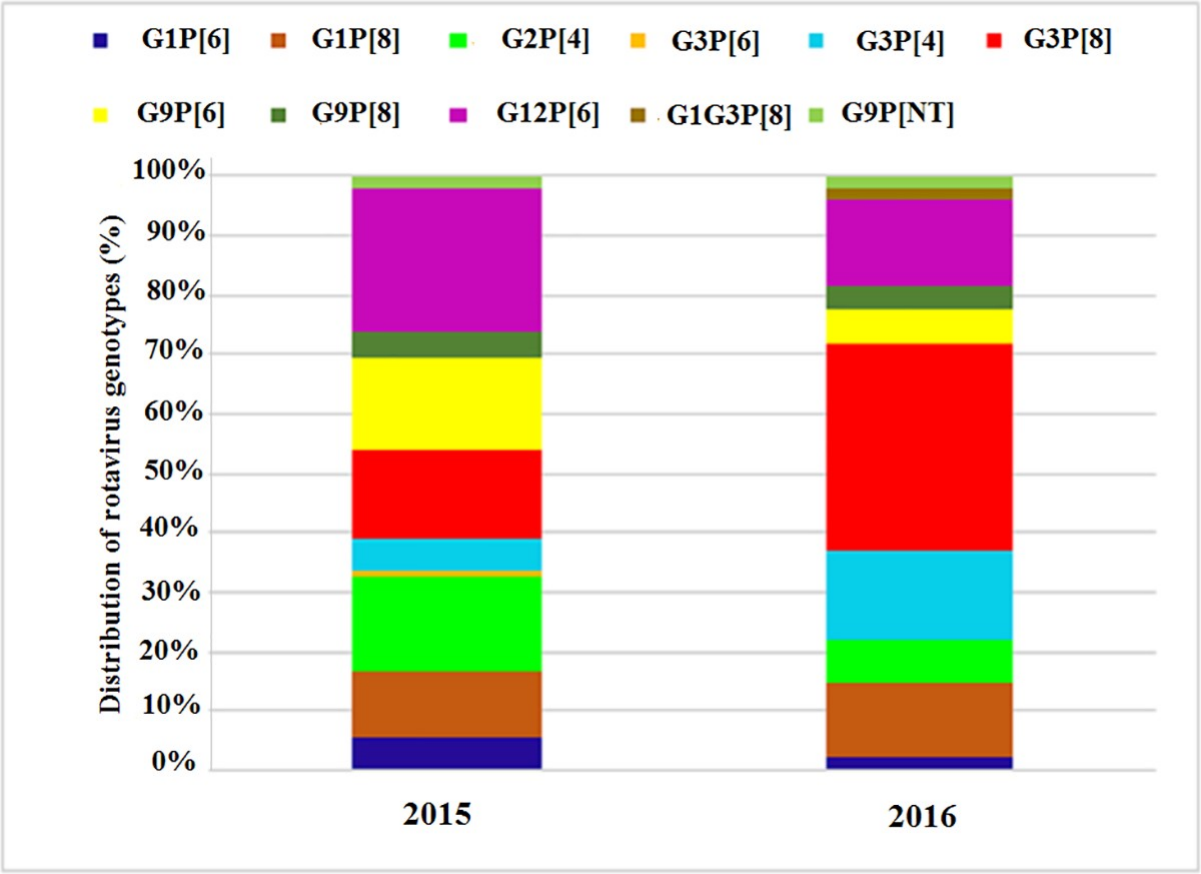
Distribution of RVA G/P genotype combinations in 2015–2016.

Annual genotypic distribution of RVA strains in 2015 to 2016 is shown in Fig 1. The most common G/P combination in 2015 was G12P[6] followed by G2P[4], G9P[6] and G3P[8]. The frequency of RVA genotypes due to G3P[8] caused by decrease in G12P[6] from 24% to 14.8% in 2016. (Fig 1).

### Seasonality

**Fig 2:**
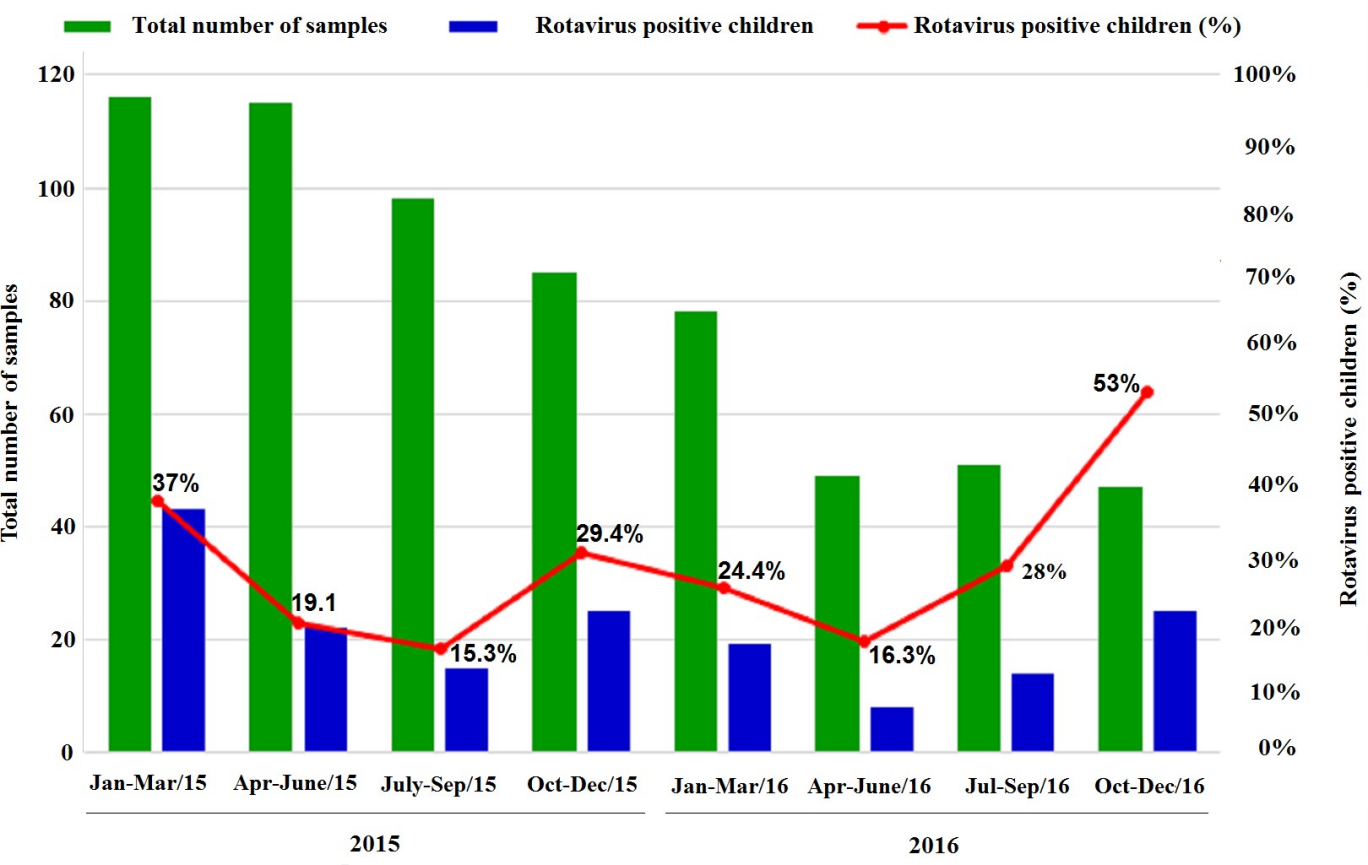
Month-wise screening of human Group A rotavirus samples among children detected with acute gastroenteritis (AGE) during 2015–2016.

RVA infections were present throughout the year over the period of the two years of surveillance (2015–2016), with highest prevalence in dry winter season. During 2015, the highest number of RVA positive cases (37%) were observed in dry winter months (January-March, 37%) of the year followed by (October-December, 29.4%). Similarly, RVA infections peaked with 53% positive cases in dry winter months (October-December) of the year 2016(Fig 2).

### Phylogenetic analysis

#### Phylogenetic analysis of gene encoding VP7

Maximum likelihood trees were constructed for selected RVA strains based on partial genome sequences of VP7 gene segment. The total number of nucleotide sequences obtained for VP7 genotypes G1, G2, G3, G9 and G12 are 20, 15, 44, 24 and 30 respectively.

**Fig 3:**
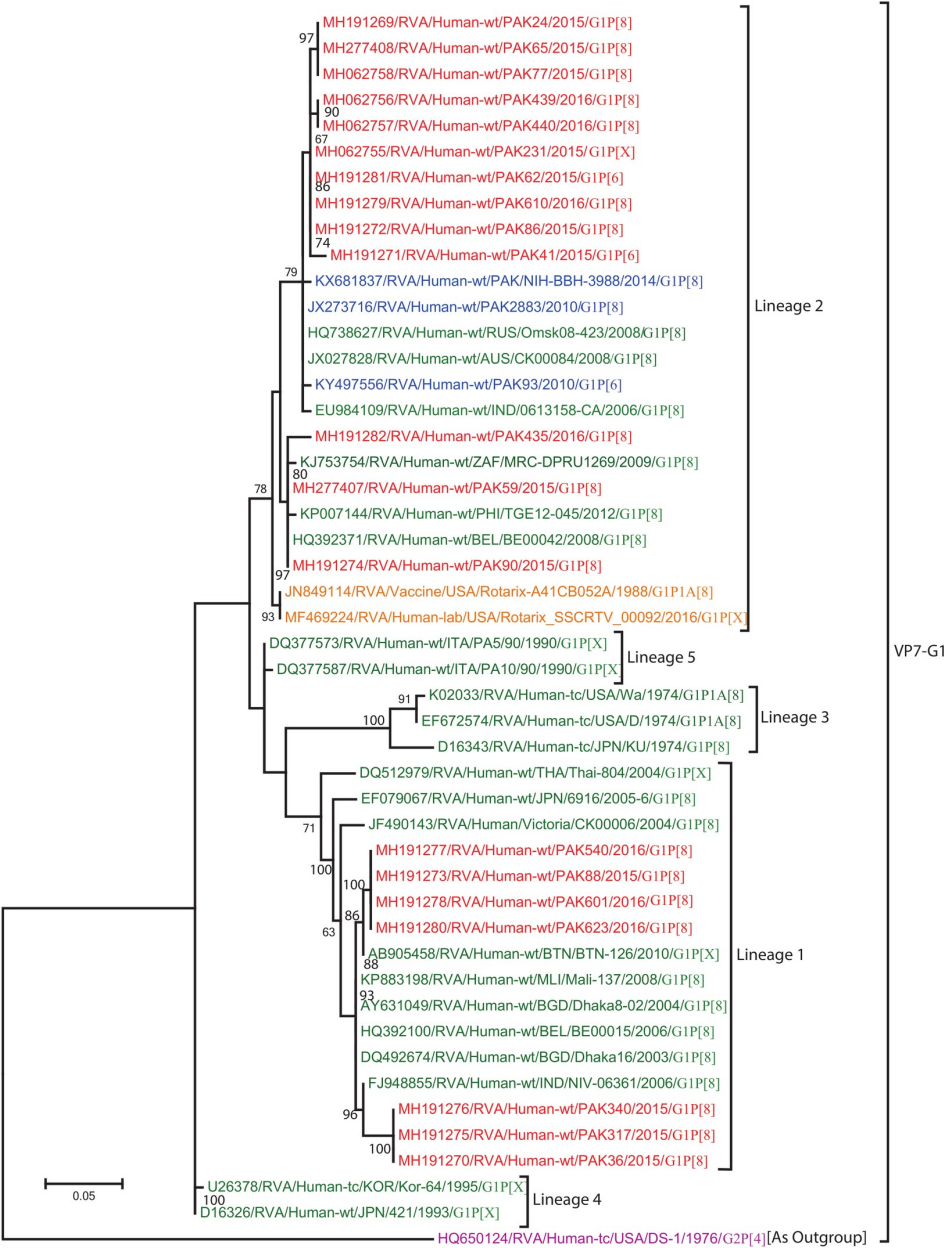
Maximum likelihood phylogenetic tree constructed from the nucleotide sequences of G1-VP7 strains and representative RVA strains with kimura-2-parameter model in mega program 6.0. **Bootstrap values <60 are not shown.** RVA strains sequenced in this study are represented by the red colour. Pakistani strains reported in the previous studies are shown in blue. The vaccine strains and an out group strain are represented by purple and orange color, respectively while green shading represent strains isolated all over the world. The RVA strains sequenced in this study and reference strains obtained from GenBank database are represented by Accession number, Strain Name, Country and year of Isolation. Scale bar: 0.05 substitutions per nucleotide.

**G1:** The G1-VP7 tree was constructed based on 20 Pakistani G1 strains sequenced in this study and representative members of the G1 genotype (Fig 3). Ten Pakistani strains (PAK24, PAK41, PAK62, PAK65, PAK77, PAK86, PAK231, PAK439, PAK440 and PAK610) are closely related to each other and cluster in lineage 2. This lineage also contained G1 strains detected in Australia (CK00084), Russia (Omsk08-423) and India (0613158-CA), as well as with three Pakistani strains (NIH-BBH-3988, PAK2883 and PAK93) detected in previous studies [23,24], with a very high nucleotide similarities (98.1–99.5%). Another subcluster in lineage 2 contained Pakistani strains PAK59, PAK90 and PAK435, closely related to strains identified from the South Africa (MRC/DPRU-1269), Belgium (BE00042) and the Philippines (TGE12-045) (nucleotide similarity of 98.1–99.5%), distinct from the Rotarix vaccine strains (Rotarix-A41CB052A and Rotarix_SSCRTV_00092) [48]. The VP7 gene of 7 other Pakistani G1 strains (PAK317, PAK36, PAK340, PAK540, PAK88, PAK601 and PAK 623) clustered together with strains reported in India (Niv-06361), Bangladesh (Dhaka16/2003), Mali (Mali-137), Bhutan (BTN-126), and Belgium (BE00015) with a nucleotide similarity of 98.1–99.5%, into lineage 1 [49–52]. All G1 strains i.e reference strains and strains isolated in this study included in the tree diverge considerably from USA G2 strain (USA/Ds-1/1976/G2P[4]) used as an outgroup.

**Fig 4:**
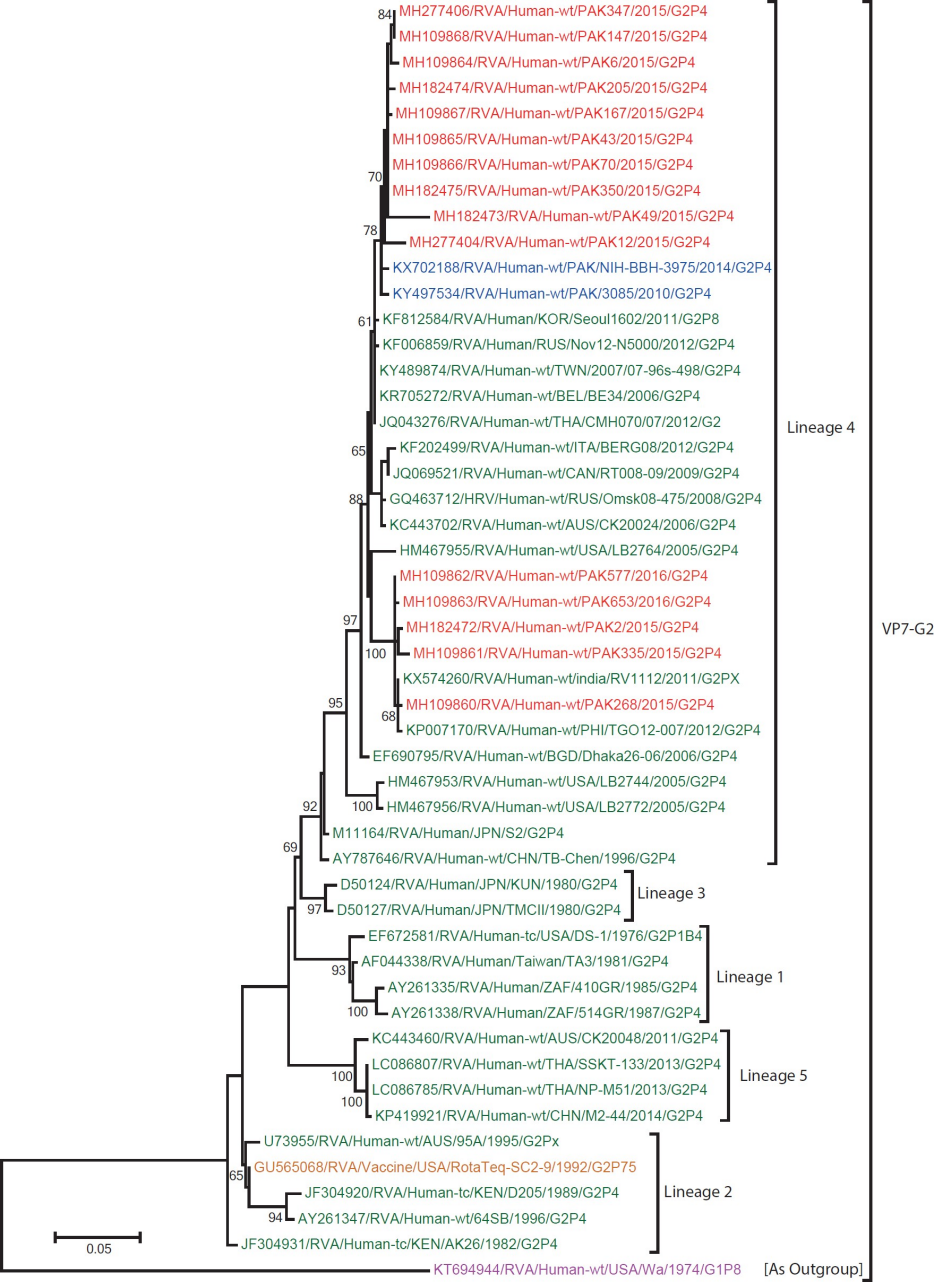
Maximum likelihood phylogenetic tree constructed from the nucleotide sequences of G2-VP7 strains and representative RVA strains with kimura-2-parameter model in MEGA program 6.0. **Bootstrap values <60 are not shown.** RVA strains sequenced in this study are represented by the red colour. Pakistani Strains reported in the previous studies are shown in blue. The vaccine strains and an out group strain are represented by purple and orange colour respectively while green shading represent strains isolated in all over the world. The RVA strains sequenced in this study and reference strains obtained from GenBank database are represented by Accession number, Strain Name, Country and year of Isolation. Scale bar: 0.05 substitutions per nucleotide.

**G2:** The G2 tree was constructed using the nucleotide sequences of 15 Pakistani G2 strains sequenced in this study and representative members of the G2 genotype (Fig 4). All Pakistani strain fell into lineage 4 [53]. Ten strains (PAK6, PAK12, PAK43, PAK49, PAK70, PAK147, PAK167, PAK205, PAK347and PAK350) were closely related to Pakistani strains NIH-BBH-3975 and PAK3085 detected in previous studies conducted in Pakistan during 2010 and 2014 [23,24], with high nucleotide similarities (98.4–99.2%). Furthermore, the clustered closely to G2 strains from Korea (Seoul1602), Russia (Nov12-N5000), Taiwan (07-96s-498), Thailand (CMH070), and Belgium (BE-34). On the other hand 5 Pakistani strains (PAK2, PAK268, PAK335, PAK653 and PAK 577) were closely related to strains from Indian (RV/1122) and the, Philippines (TG012-007) with a nucleotide identity of 97.3–99.8%. All G2 strains i.e reference strains and strains isolated in this study included in the tree diverge considerably from USA G1 strain (Wa/1974/G1P[8]) used as an outgroup.

**Fig 5:**
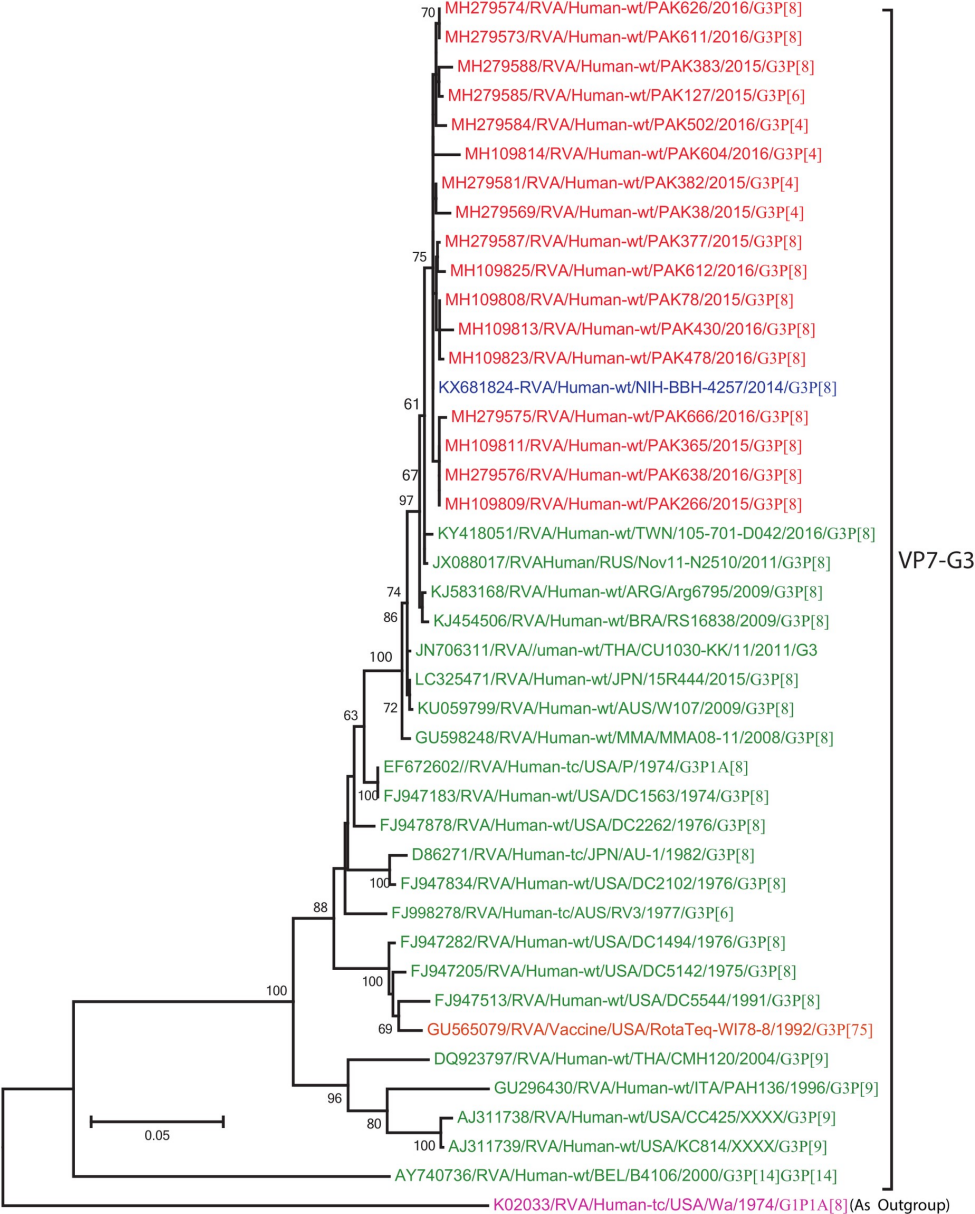
Maximum likelihood phylogenetic tree constructed from the nucleotide sequences of G3-VP7 strains and representative RVA strains with kimura-2-parameter model in MEGA program 6.0. **Bootstrap values <60 are not shown.** RVA strains sequenced in this study are represented by the red colour. Pakistani Strains reported in the previous studies are shown in blue. The vaccine strains and an out group strain are represented by purple and orange colour respectively while green shading represent strains isolated in all over the world. The RVA strains sequenced in this study and reference strains obtained from GenBank database are represented by Accession number, Strain Name, Country and year of Isolation. Scale bar: 0.05 substitutions per nucleotide.

**G3**: A total of 17 Pakistani G3 strains sequenced in this study and representative members of G3 strains were selected for phylogenetic analyses of the G3 genotype (Fig 5). All Pakistani G3 strains, including NIH-BBH-4257, previously isolated in Pakistan in 2014 [24], clustered closely together (99–100% nucleotide similarity) with each other and strains detected in Taiwan (105-701-D042), Russia (Nov11-N2510), Brazil (RS16838) and Argentina (Arg6795). All G3 strains i.e reference strains and strains isolated in this study included in the tree diverge considerably from USA G1 strain (Wa/1974/G1P[8]) used as an outgroup.

**Fig 6:**
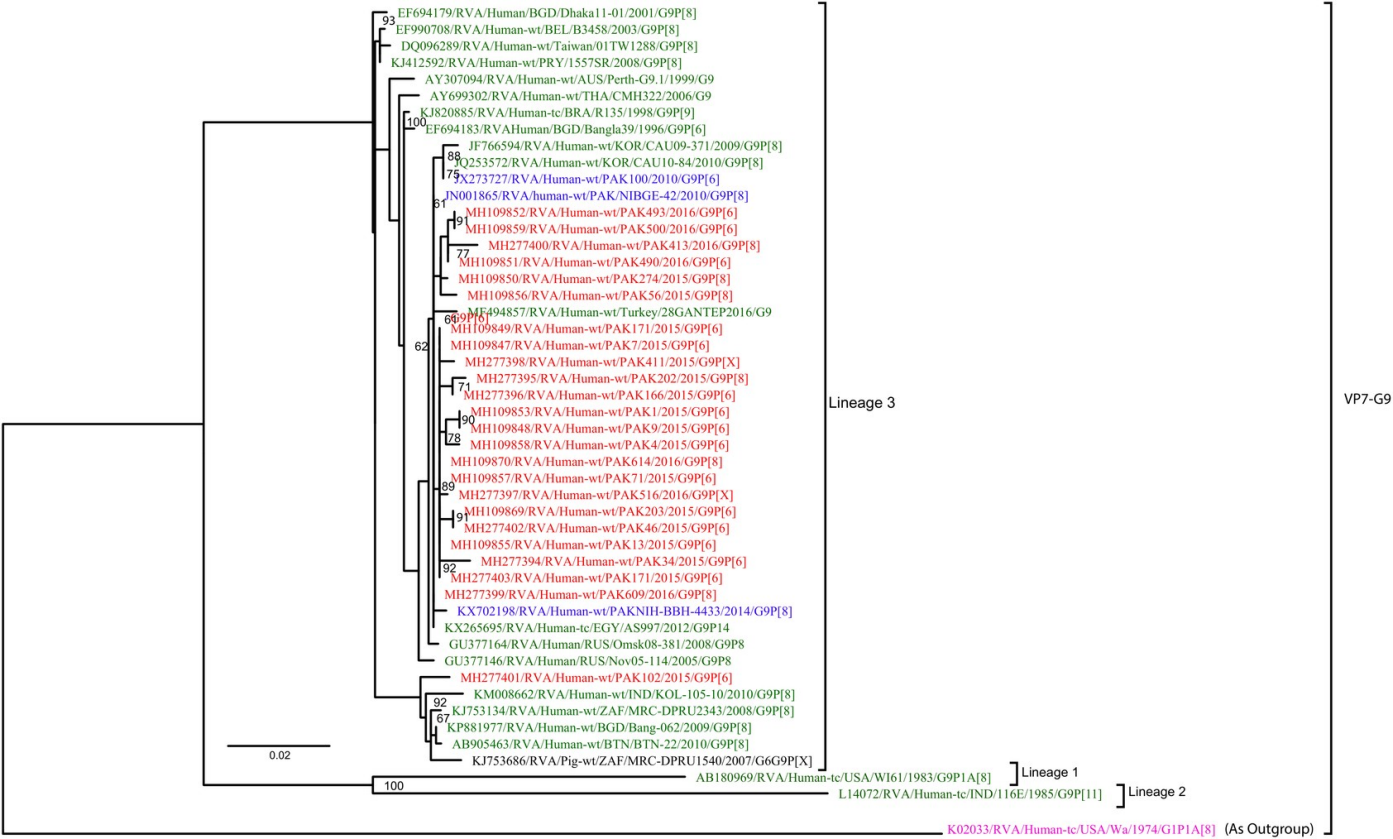
Maximum likelihood phylogenetic tree constructed from the nucleotide sequences of G9-VP7 strains and representative RVA strains with kimura-2-parameter model in MEGA program 6.0. **Bootstrap values <60 are not shown.** RVA strains sequenced in this study are represented by the red colour. Pakistani Strains reported in the previous studies are shown in blue. The out group strain is represented by purple colour, Pig strain is represented by Black Colour while green shading represent strains isolated in all over the world. Scale bar: 0.02 substitutions per nucleotide. The RVA strains sequenced in this study and reference strains obtained from GenBank database are represented by Accession number, Strain Name, Country and year of Isolation. Scale bar: 0.02 substitutions per nucleotide.

**G9:** A total of 24 Pakistani G9 strains sequenced in this study and representative members of the G9 genotype were selected for the phylogenetic analyses (Fig 6). All Pakistani strain fell into G9 lineage 3. Twenty-tree of these G9 strains showed a high nucleotide identity (99–100%) with other strains detected in Pakistan (PAK100, NIBGE-42 and NIH-BBH-4433) in 2010 and 2014 [23,24], as well as with other strains circulating in Turkey (28GENTEP), Russia (omsk08-381, Nov5-114), Egypt (AS997), and Korea (CAU09-371 and CAU10-84). On the other hand PAK102 showed 98.7% nucleotide similarity with the South African pig rotavirus strains MRC-DPRU1540 and human RVA strains from India (Kol-105-10), Africa (ZAF/MRC-DPRU2343), Bhutan (BTN-22) and Bangladesh (Bang-062). All G9 strains i.e reference strains and strains isolated in this study included in the tree diverge considerably from USA G1 strain (USA/Wa/1974/G1P[8]) used as an outgroup.

**Fig 7:**
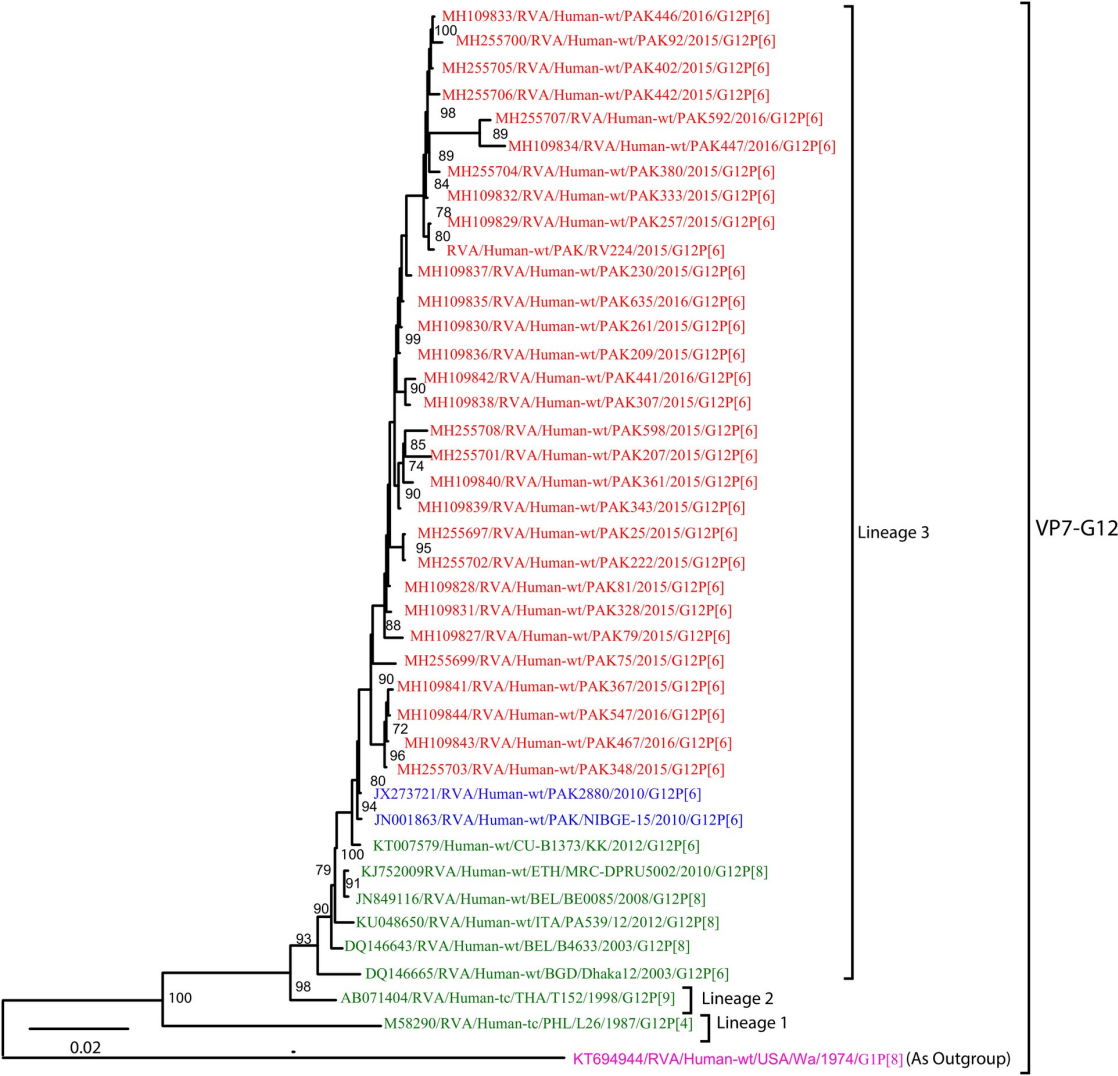
Maximum likelihood phylogenetic tree constructed from the nucleotide sequences of G12-VP7 strains and representative RVA strains with kimura-2-parameter model in MEGA program 6.0. **Bootstrap values <60 are not shown.** RVA strains sequenced in this study are represented by the red colour. Pakistani Strains reported in the previous studies are shown in blue. The out group strain is represented by purple colour while green shading represent strains isolated in all over the world. The RVA strains sequenced in this study and reference strains obtained from GenBank database are represented by Accession number, Strain Name, Country and year of Isolation. Scale bar: 0.02 substitutions per nucleotide.

**Fig 8:**
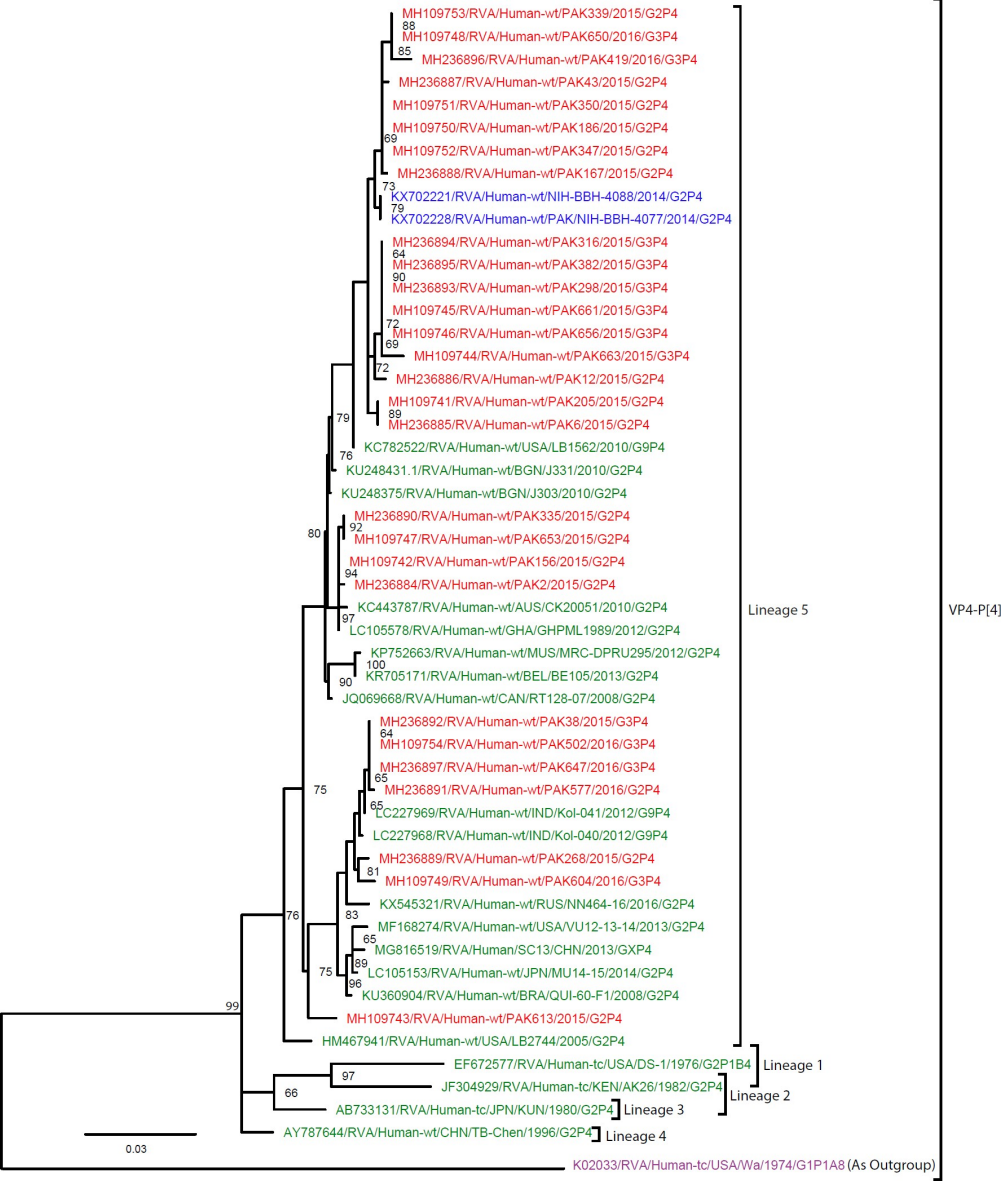
Maximum likelihood phylogenetic tree constructed from the nucleotide sequences of P[4]-VP4 strains and representative RVA strains with kimura-2-parameter model in MEGA program 6.0. **Bootstrap values <60 are not shown.** RVA strains sequenced in this study are represented by the red colour. Pakistani Strains reported in the previous studies are shown in blue. The out group strain is represented by purple colour while green shading represent strains isolated in all over the world. The RVA strains sequenced in this study and reference strains obtained from GenBank database are represented by Accession number, Strain Name, Country and year of Isolation. Scale bar: 0.03 substitutions per nucleotide. Scale bar: 0.03 substitutions per nucleotide.

**G12:** A total 30 Pakistani G12 strains sequenced in this study and representative members of the RVA G12 genotype were included in the construction of VP7-G12 phylogenetic tree (Fig 7). All Pakistani G12 strains belong to lineage 3 and are closely related to each other with 99–100% nucleotide similarity, clustering with other strains isolated in Bangkok (CU-B1373), Ethiopia (MRC-DPRU5002), Belgium (B4633), Italy (PA539), Bangladesh (Dhaka/12/2003), Thailand (T152), Philippine (L26) and with two Pakistani strains (PAK2880, NIBGE-15) isolated in previous studies during the years 2010 and 2014 (Fig 8). All G12 strains i.e reference strains and strains isolated in this study included in the tree diverge considerably from USA G1 strain (Wa/1974/G1P[8]) used as an outgroup.

#### Phylogenetic analysis of gene encoding VP4

Maximum likelihood trees were constructed for selected partial genome sequences based on gene segment VP4. The total number of nucleotide sequences obtained for VP4 genotypes P[4], P[6] and P[8] are 28, 51 and 51 respectively.

**P[4]:** A total of 28 P[4] strains sequenced in this study and representative members of P[4] genotype were selected for the construction of VP[4] phylogenetic tree (Fig 8), showing that all Pakistani strains belonged to VP4-lineage 5. A clusters of 17 P[4] strains was closely related (99–100% nucleotide similarity) with two Pakistani strains (NIH-BBH-4088, NIH-BBH-4077) isolated in previous studies in 2014 and with another strains circulating in the USA in 2010 (LB-1562). One cluster of four Pakistani Strains (PAK2, PAK156, PAK335, and PAK653) showed close nucleotide identity (99.8–100%) with each other and other strains detected in Australia (CK20051) and Ghana (GHPML1989). Another clusters of six P[4] strains (PAK577, PAK647, PAK502, PAK38, PAK268 and PAK604) showed 99.4–100% nucleotide similarity with each other and form a cluster with P[4] strain detected in India (Kol-040 *and Kol-041) and Russia (NN464-16/)*. Finally, a single strain PAK613 formed a separate cluster, most closely related (97.3–97.5% nt similarity) with strains circulating in India, Russia, USA, China, Japan and Brazil and other Pakistani strains isolated in this study. All P4 strains i.e reference strains and strains isolated in this study included in the tree diverge considerably from USA G1 strain (Wa/1974/G1P[8]) used as an outgroup.

**Fig 9:**
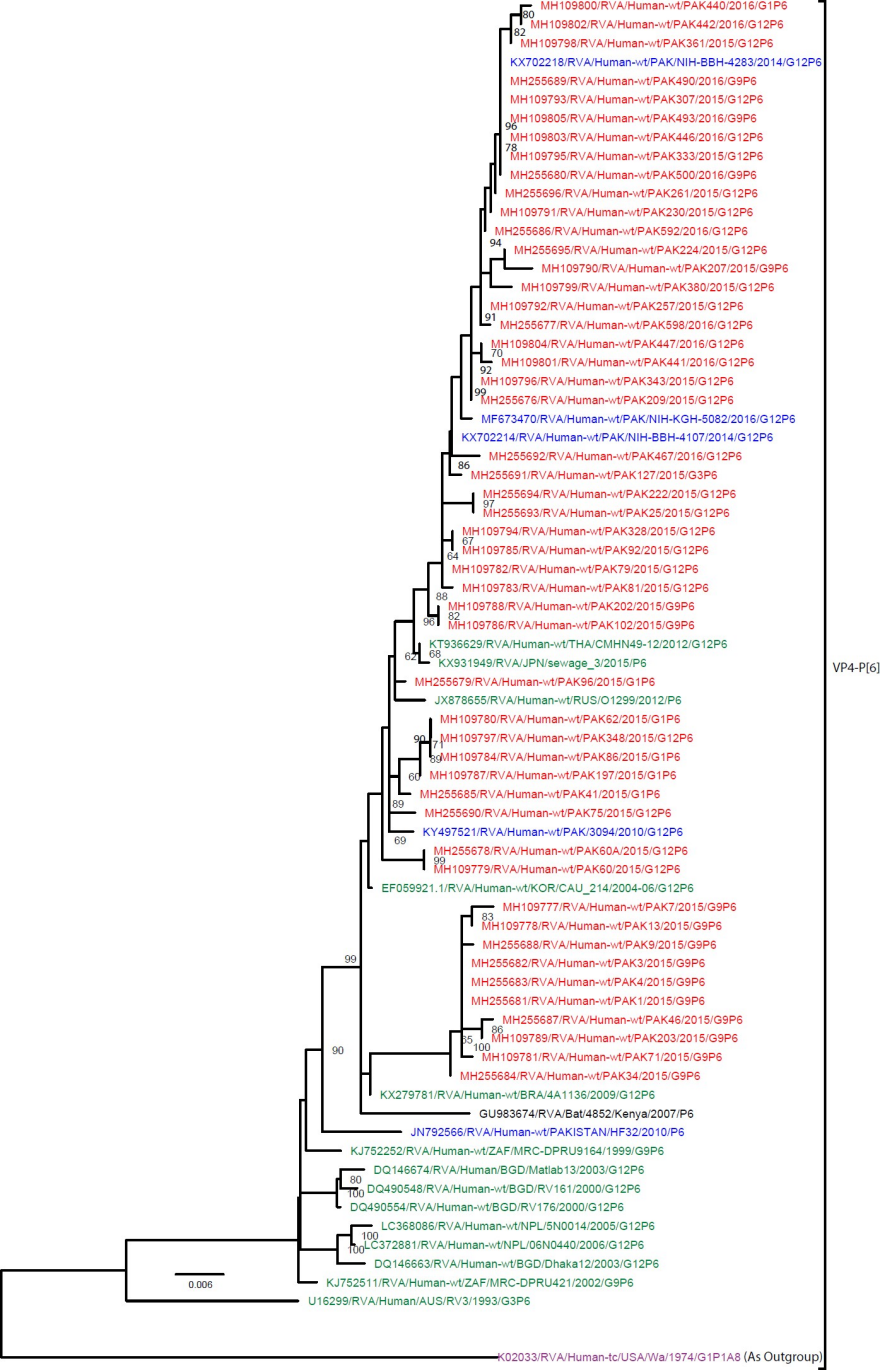
Maximum likelihood phylogenetic tree constructed from the nucleotide sequences of P[6]-VP4 strains and representative RVA strains with kimura-2-parameter model in MEGA program 6.0. **Bootstrap values <60 are not shown.** RVA strains sequenced in this study are represented by the red colour. Pakistani Strains reported in the previous studies are shown in blue. The out group strain is represented by purple colour, Bat RVA strain is represented by Black Colour while green shading represent strains isolated in all over the world. The RVA strains sequenced in this study and reference strains obtained from GenBank database are represented by Accession number, Strain Name, Country and year of Isolation. Scale bar: 0.006 substitutions per nucleotide.

**P[6]:** A total of 50 Pakistani P[6] strains sequenced in this study and representative members of the P[6] genotype were selected for the construction of P[6] phylogenetic tree (Fig 9). One cluster of 40 PAK P[6] strains showing (98.2–99.8%) nucleotide similarity with five Pakistani strains (NIH-BBH-4283, NIH-BBH-4107, NIH-KGH-5082 and PAK3094) detected in previous studies and with other strains isolated in Thailand (CMHN49-12), Russia (O1299), Japan(sewage_3) and Korea(CAU_214). Similarly one more clusters of ten PAK P[6] strains (PAK7, PAK13, PAK9, PAK3, PAK4, PAK1, PAK46, PAK203, PAK71 and PAK34) are similar to each other at (98.5–100%) nucleotide similarity level and on phylogenetic analysis making cluster with other strains circulating in Brazil (4A1136), South Africa (ZAF/MRC-DPRU9164 and ZAF/MRC-DPRU421), Bangladesh (Matlab13, RV176 and Dhaka12/2003), Australia (RV3) and one Pakistani strain (HF32) detected in previous hospital based study in 2013. Of note, a single P[6] bat rotavirus strain Bat/4852 detected in Kenya in 2007 [54], was also found in this P[6] cluster. All P6 strains i.e reference strains and strains isolated in this study included in the tree diverge considerably from USA G1 strain (Wa/1974/G1P[8]) used as an outgroup.

**Fig 10:**
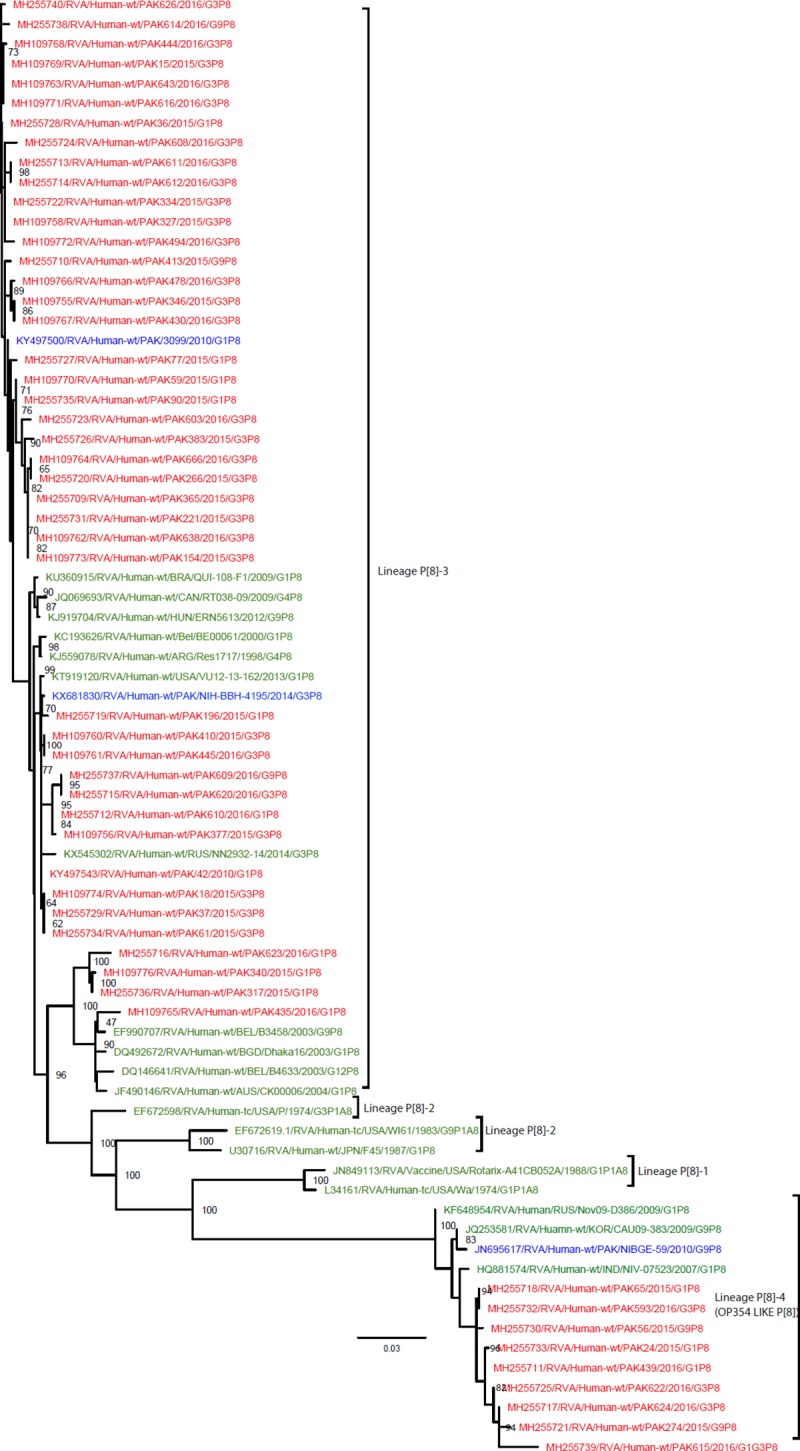
Maximum likelihood phylogenetic tree constructed from the nucleotide sequences of P[8]-VP4 strains and representative RVA strains with kimura-2-parameter model in MEGA program 6.0. **Bootstrap values <60 are not shown.** RVA strains sequenced in this study are represented by the red colour. Pakistani Strains reported in the previous studies are shown in blue. The out group strain is represented by purple colour while green shading represent strains isolated in all over the world. The RVA strains sequenced in this study and reference strains obtained from Genbank data library are represented by Accession number, Strain Name, Country and year of Isolation. Scale bar: 0.03 substitutions per nucleotide.

**P[8]:** A total of 52 Pakistani P[8] Strains sequenced in this study and representative members of P[8] genotype were selected for the construction of P[8] phylogenetic tree (Fig 10). Most of the circulating strain (n = 43) fell into P[8] lineage 3. One subcluster of 39 Pakistani P[8] strains showed 97.1–100% nt similarity with each other and clustered with strains detected in Brazil (QUI-108-F1), Argentina (Res1717), Belgium (BE00061), Canada (RT038-09), Hungry (ERN5613), USA(VU12-13-162), Russia (NN-2932-14) and previously detected P[8] strains from Pakistan (NIH-BBH-4195 and PAK3099) [23,24]. Another lineage 3 subcluster (97.1–100% nt similarity) contained four Pakistani P[8] strains (PAK623, PAK340, PAK317 and PAK435) as well as strains detected in Belgium (B3458 and B4463), Bangladesh (Dhaka16/2003) and Australia (CK0006). Interestingly another cluster of nine Pakistani P[8] strains (PAK65, PAK593, PAK56, PAK24, PAK439, PAK 622PAK624, PAK274) clustered closely with the divergent OP354-like lineage (lineage 4) strains together with strains detected in Russia (Nov09-D386), India (NIV-07523), Korea (CAU09-383) and one previously detected strain from Pakistan (NIBGE-59) during 2010.

## Discussion

RVA gastroenteritis is considered to be a leading cause of infant and childhood morbidity and mortality, particularly in developing countries like Pakistan [55]. The current study reported the circulating genotypes of human RVA during a two years surveillance activity (2015 and 2016) in Rawalpindi and Islamabad, Pakistan.

The prevalence of RVA found in the current study was 27% which is comparable with the results of previous studies (29–34%) reported from Pakistan during 2008–2014 [23,25,27,30,31]. However, a higher percentage of RVA infections was also reported in the past from Karachi and Faisalabad (57% and 63%, respectively) [28,29]. On the other hand, a community based study by Qazi and colleagues in Karachi Pakistan found a RVA prevalence of 17% [27], which is much lower as compared to current study. There might be several reasons for these observed prevalence differences, such as a different study design, the sampling period (throughout the year or during rotavirus peak season or epidemics), type of health facilities in target hospital, type of used diagnostic procedures, patients age criteria and patient testing criteria. The RVA prevalence in our study was lower when compared with studies reported in other countries like India, Cambodia, Thailand, Mongolia [56–59] and was high when compared with china [60].

The RT-PCR assay failed to detect RVA in 28 faecal samples positive by ELISA. This could be due to one or more of the following reasons; i) false positive ELISA result, ii) PCR inhibitors that cause positive samples to be negative by PCR, iii) drop of the viral titer below PCR detection limit due to additional shipment and freeze-thaw cycles.

There are seasonal and geographical variations in RVA based infections globally. In temperate climates, RVA epidemics commonly appear during the dry, winter months of the year. However, in most tropical areas, RVA gastroenteritis is prevalent throughout the year without seasonal fluctuation [61]. In the current study RVA gastroenteritis is seen throughout the year with highest detection rate in winter months of the year. The results of the present study are similar to previous studies from Pakistan which have reported RVA infections throughout the year with dominant peaks in winter and dry months of the year [23–25,27]. The same patterns have also been reported in other Asian countries like Bangladesh, India and Thailand [62–64].

In the present study, no gender differences were found in RVA infections, which is in accordance with the previous reported cases from Pakistan [24,27]. A large proportion (71.4%) of children with RVA gastroenteritis is in less than 1 year of age which is also in agreement with previous work from Pakistan [23,24,27,28]. Some studies have revealed that children younger than 4 months are protected against severe RVA diarrhea mainly due to maternal antibodies and breast feeding which may be the reason for the higher RVA incidence after 7 months of age [65,66]. A low incidence (3.5%) of RVA infection is also reported in older children (>5 years) in this study, an age group in which RVA infections are only poorly appreciated worldwide. The similar trends has also been documented in previous studies reported in Pakistan, Angola, Kenya, Nepal, India, Turkey, China and Bangladesh [16,23,27,60,67–72]. In adults, RVA infections can be symptomless or can be accompanied by nausea, malaise, headache, abdominal pain, fever and diarrhea. In immunocompromised adults, it can range from symptomless to chronic infection [73].

The rotavirus molecular epidemiology varies from country to country, depending upon the socio-economic status and weather conditions [74]. The most common G-genotypes affecting humans are G1, G2, G3, G4, G9 and G12 while the most common human P-genotypes are P[4], P[6] and P[8] [75,76]. The 6 main epidemiologically significant genotypes combinations prevailing around the globe are G1P[8], G2P[4], G3P[8], G4P[8], G9P[8] and G12P[8] [77,78]. In previous studies, the four most prevalent G genotypes reported in Pakistan were G1, G2, G3 and G9, while the three most common P types were P[4], P[6] and P[8] [23,25–29,31,79]. The genotype G3P[8] with G2P[4] and G12P[6] were found to be the dominant genotypes in 2015 and 2016.

The detection rate of the G1 genotype in the current study is 16% which is in accordance with two previous hospital based studies (11.6% and 14.5%) conducted during the years 2008 and 2014, respectively [24,27]. However, this prevalence rate was lower than a study conducted by Tamim and colleagues who reported that 24.3% of the infection were cause by G1 strains during the year 2010 [23]. G1 is responsible for 70% of infections in Europe, North America and Australia, while in south America, Africa and Asia it accounts for 20–30% infections [70]. Phylogenetic analysis suggested that, Pakistani G1 strains isolated during current study clustered with other strains detected worldwide into two distinct G1 Lineages (Lineage 1 and 2, Fig 3). The different G1 lineages possess antigenic variation at both amino acid and nucleotide level [80].

In this study the G2 genotype is reported in 13% of the cases, which is less than the previous studies (19–24%) reported during 2010 and 2014 [23,24]. Phylogenetic analysis showed that all Pakistani G2 strains from this study clustered in lineage 4 with reference strains from USA, India, Bangladesh, Russia, Canada, Korea, Philippines, Belgium, Italy, Taiwan and two locally circulating strains identified in previous studies (Fig 4).

The G3 genotype is recognized as the third most predominant RVA genotype in humans mostly found in combination with P[8] [81]. RotaTeq, a pentavalent human bovine reassortment vaccine contain G3P[8] genotype in its formulation [82]. Although no direct causal link has been proven, G3 strains with different P types P[4], P[6], P[8] and P[9] have been detected in humans round the world after introduction of RVA vaccines in immunization program of many countries [83,84]. In Pakistan G3 genotype was first reported in 2010 in Faisalabad region in very low percentage (2.6%) [28]. In the present study the G3 was detected with the P[4], P[6] and P[8] genotypes. G3P[8] was found to be the dominant genotype in this study which is similar to a previous study conducted by Umair et al in 2014 [24]. In recent years, G3P[8] has emerged as a predominant genotype in Asia, Europe and South America. These newly discovered viral strains showed high similarities with equine-like G3P[8] strains and are able to spread rapidly in the human population [85,86,87,88]. The introduction of rotavirus mass vaccination in many countries of the world might be the reason for the evolution of new variant genotypes. The emergence of G3P[8] as a dominant strain in Pakistan in 2016 was more or less coinciding with the import of new variant G3P[8] strains from other countries to Pakistan.

The human G9 RVA genotype has gained epidemiological significance since mid-1990s and is now acknowledged as the fifth main human RVA genotype [89]. According to an WHO report in 2013, G9 is most frequently found in combination with the P[8] genotype as is less prevalent with the P[4], P[6], P[11] and P[19] genotypes [90,91]. The G9 genotype accounts for 7.4% RVA infections globally and is more commonly present in south Asia and Middle East (20.3%) and is less prevalent in Southeast Asia (5.9%) [92]. Many studies have revealed a close genetic relationship between human and Pig G9 RVA strains, suggesting that interspecies transmissions in combination with reassortment has resulted in the emergence of this genotype in humans [93–95]. In Asia, G9 RVA strains are the second most abundant genotype in pigs with a detection rate of 11.4% [96]. In the present study the G9 prevalence rate is 18% which is more than the previously reported G9 rates in Pakistan [23–25,27]. The G9 isolates detected in this study found in combination with P[6] and P[8] genotypes. G9P[6] was more prevalent (11.9%) than G9P[8] (4.2%). These G9 strains are showing close sequence similarity, suggesting that the frequent reassortment events have occurred. Pakistani G9 RVA strains have clustered very close to each other in lineage 3 with other strains isolated all over the world. One Pakistani G9 strain (PAK102) is showing 98.7 nucleotide identity with a porcine RVA strain found in South Africa (RVA/Pig-wt/ZAF/MRC-DPRU1540/2007/G6G9PX). This sequence was directly submitted to GenBank and was identified in a mixed infected sample [97].

The first G12 genotype (G12P[4]) was discovered in 1987 from the Philippines, in children less than 2 years old, affected with acute diarrhea [98,99]. A decade later, G12 was identified as an emerging genotype in several countries of Asia, Europe, North America and South America mostly in combination with P[8] and P[6] [78,100,101]. Now, G12 has been spreading as a devastating genotype in many Asian countries including Pakistan [102]. These unusual strains such as G12P[6] are not included in the formulation of two available rotavirus vaccines Rotarix (GlaxoSmithkline) and RotaTeq (Merck). Hence, it has not yet been determined, whether or not these two available vaccines provide protective immunity against these unusual strains [102]. The continuous monitoring and whole genome-based analyses are essential in understanding the evolutionary dynamics and the spread of G12 strains in Asia. In Pakistan, G12 strains were reported by Alam and colleagues in two children of Rawalpindi, Pakistan for the first time in 2009 [103]. Other studies from the same region by Tamim in 2010 and Umair in 2014 detected G12 with 6.7% and 16.7% prevalence, respectively [23,24]. In the present study genotype G12P[6] was found as the second most abundant genotype with 21% prevalence in Pakistan. This emphasises the continuous spread of the G12 genotype in Pakistan from 2009 to 2016, as well as in Asian countries. On phylogenetic observation, Pakistani G12 strains were most closely associated with Thailand strain (B1373). This reflects the close connection with this country in terms of travel and other business activities [104,105]. The high prevalence of G12 genotype during the same period was also detected in neighbouring country Bangladesh [62]. Therefore, it is suggested that the emergence of G12 in 2015 as a dominant strain in Pakistan more likely was due to their spread from other Asian countries to Pakistan.

The dominance of G3P[8] genotype in 2014, G12P[6] genotype in 2015 and further re-emergence of G3P[8] as a dominant genotype in 2016 in Pakistan in the current study suggested that the G3 and G12 strains might be competing and excluding one another in the same vulnerable population. Moreover, the increasing implementation of RVA mass vaccination worldwide might be the reason for genotypic fluctuations and emergence of new genotypes that lead to their global spread including Pakistan.

The P[4] genotype of human RVAs is mostly identified with VP7 genotypes G2 and G8 [106]. However, in the present study P[4] is found in combination with G2 and G3, and is the third most abundant genotype found in 22% of study cases. Phylogenetic analysis of the P[4] genotype revealed that all Pakistani P[4] strains fall in lineage 5 with other strains isolated from USA, India, Bangladesh, Japan, Russia, China, Canada, Ghana, Belgium, Brazil and previously identified Pakistani strains (Fig 8).

The RVA P[6] genotype is detected all over the world with many G genotypes (G1-G6, G8-G12, and G25) [107,108]. In humans the P[6] genotype is considered to be the most prevalent genotype in South Asia and Sub-Saharan Africa [109]. In the present study P[6] is the second most dominant (37%) P-genotype found in the Pakistani population. Comparison of RVA genotype P[6] with reference strains from BLAST search reported similarity with human as well as with one bat RVA strain from Kenya (RVA/Bat-wt/KEN/KE4852/2007/G25P[6]), although it is more likely that the bat P[6] was derived from humans, rather than the other way around [110].

A distinct subtype of the P[8] genotype, P[8]-4 or OP354-like P[8] has been detected in many countries of Europe, Africa and Asia [111]. According to Zeller and colleagues, South and East Asia are the main origin from where OP354-like P[8] strains migrated to Africa, Europe, and North America [112]. In the present study, the P[8] genotype is found to be dominant (39%), and based on our phylogenetic analysis nine Pakistani strains possess an OP354- like P[8], whereas all other strains are closely related to the P[8] strains revolving in other countries of the world in most common P[8] Lineage 3. These results show two distinct P[8] lineages are co-circulating in Pakistan at the same time. Owing to antigenic differences in the VP4 spike of the OP354-like strain, it has been questioned whether or not the P[8] moiety in the vaccines would equally well protect against this divergent variant [113].

RVA mixed infection rate has increased globally from 7.9–11.7% from 1997 to 2007 [9,114]. The RVA mixed infection rate observed in the present study is 0.7% which is similar to previous reports [23,24,26–28]. The total of 2.4% of the study samples were P-Non-typeable even with different primer sets. The possible reasons for genotyping failure might be the primer mismatch due to higher RVA diversity.

The unusual genotypes (G1P[6], G3P[4] and G9P[6]) accounted for 25% of total RVA genotypes in this study, which is lower than previous data reported by Masab and colleagues [24]. The unusual genotypes and mixed genotypes arise due to mixed infections that lead to the reassortment and evolution of novel genotypes. Among unusual genotypes, G3P[4] acquire attention, as it is the second time that G3 in combination with P[4] is reported in Pakistan in this study. This genotype emerged most likely after an intergenogroup re-assortment in which G2P[4] strain already circulating in population may have acquired VP7 gene from locally co-circulating G3 human RVA strains. Likewise already circulating G3P[8] and G3P[6] strains in population can be the parental strains of G3P[4] reassortant strain. Similarly OP354-like P[8] strains detected in the present study were found in combination with G1, G3 and G9. This suggests that there is a frequent ongoing reassortment phenomenon in progress.

Pakistan is one of the countries with the highest disease burden of RVA infections in children of less than five years of age. The introduction of two RVA vaccines has reduced the global mortality rate and number of hospitalizations in many countries [10,14,115]. Pakistan has completed phased introduction of RVA vaccines in the national EPI program in January 2018. The hospital based studies regarding efficacy of RVA vaccines and its effects on the country’s specific strains are yet to be determined [23,24,26,27]. However, in a recent survey conducted by Richard and his colleagues, Sindh and Baluchistan province of Pakistan bear the highest RVA burden which is estimated to be 3.1–3.3 rotavirus deaths/1000 births. The vaccine coverage in these two provinces is estimated to be lowest, particularly in children living in the poorest households conditions. Overall, RVA vaccine introduction in Pakistan could prevent 3061 deaths per year with current routine immunization program with highest coverage in capital city Islamabad that could prevent an additional 1648 deaths per year [116].

However, we believe that with the inclusion of more samples for sustained time period and recruiting multiple sentinel sites throughout the country will be required for better understanding the scale of problem. Furthermore, whole genome characterization of human RVA strains and inclusion of samples from animal and environmental sources in future studies is recommended to monitor the ongoing interspecies transmission and reassortment events.

## Conclusions

This study concludes the high prevalence of G12 and G3 RVA strains in Pakistan in 2015 and 2016 in Rawalpindi and Islamabad, Pakistan. Although it provides an incomplete epidemiological picture of gastroenteritis caused by RVA throughout the country, the results of this study will provide comprehensive knowledge to the researchers and public health authorities to calculate the RVA disease burden in the country. The observed large diversity of RVA genotypes and their yearly fluctuations demand an intensive, large scale surveillance system in the country. This will help to monitor evolving of unusual RVA genotypes and to assess the impact of vaccine introduction on these recently emerged RVA strains in Pakistani population.

## Supporting information

S1 Table GenBank accession numbers assigned for all rotavirus genotypes based on gene segment VP4 and VP7 sequenced in this study. (DOCX)

S1 Fig Identification of rotavirus A VP7 gene segment by using Beg9 and End 9 primers. Lane 1: positive control, Lane 2: DNA ladder 50 bp, lane 3, 4, 5, 6, 8, 9, 10, 11, 13: Amplified product of gene segment VP7(1062 bp size) of samples no 1, 2, 3, 4, 6, 7, 9, 12 and 15, Lane7, 12: Samples number 5, 13 negative for VP7 gene, Lane 14, 15: Negative control and non-target control(NT-C), respectively.
(TIF)

S2 Fig Identification of rotavirus A VP4 gene segment by using VP4 1-17F and Con2 primers. Lane 1: DNA ladder 50 bp, lane 2, 3, 4, 5, 6, 8: Amplified product of gene segment VP4 (876 bp size) of samples no 1, 2, 3, 4, 6 and 16, Lane7: Samples number 13 negative for VP4 gene, Lane 9, 10 and 11: Positive control, Negative control and non-target control (NT-C), respectively.
(TIF)
