# Supplementary material for: Molecular characterization of human group A rotavirus genotypes circulating in Rawalpindi, Islamabad, Pakistan during 2015-2016
Source: PLoS One. 2019 Jul 30;14(7):e0220387. doi: 10.1371/journal.pone.0220387 (PMC6667158; doi:10.1371/journal.pone.0220387)
Supplement: S1 Table — (DOCX) [file pone.0220387.s001.docx]

**Table S1. GenBank accession numbers assigned for rotavirus genotypes based on gene segment VP4 and VP7**

**sequenced in this study**

| **Strain Name** | **Accession Number** | **Strain Name** | **Accession Number** |
| --- | --- | --- | --- |
| **VP4 genotypes** | | | |
| **P[4]** | | | |
| RVA/Human-wt/PAK2/2015/G2P4 | MH236884 | RVA/Human-wt/PAK347/2015/G2P4 | MH109752 |
| RVA/Human-wt/PAK6/2015/G2P4 | MH236885 | RVA/Human-wt/PAK350/2015/G2P4 | MH109751 |
| RVA/Human-wt/PAK12/2015/G2P4 | MH236886 | RVA/Human-wt/PAK382/2015/G3P4 | MH236895 |
| RVA/Human-wt/PAK38/2015/G3P4 | MH236892 | RVA/Human-wt/PAK419/2016/G3P4 | MH236896 |
| RVA/Human-wt/PAK43/2015/G2P4 | MH236887 | RVA/Human-wt/PAK502/2016/G3P4 | MH109754 |
| RVA/Human-wt/PAK156/2015/G2P4 | MH109742 | RVA/Human-wt/PAK577/2016/G2P4 | MH236891 |
| RVA/Human-wt/PAK167/2015/G2P4 | MH236888 | RVA/Human-wt/PAK604/2016/G3P4 | MH109749 |
| RVA/Human-wt/PAK186/2015/G2P4 | MH109750 | RVA/Human-wt/PAK613/2015/G2P4 | MH109743 |
| RVA/Human-wt/PAK205/2015/G2P4 | MH109741 | RVA/Human-wt/PAK647/2016/G3P4 | MH236897 |
| RVA/Human-wt/PAK268/2015/G2P4 | MH236889 | RVA/Human-wt/PAK650/2016/G3P4 | MH109748 |
| RVA/Human-wt/PAK298/2015/G3P4 | MH236893 | RVA/Human-wt/PAK653/2015/G2P4 | MH109747 |
| RVA/Human-wt/PAK316/2015/G3P4 | MH236894 | RVA/Human-wt/PAK656/2015/G3P4 | MH109746 |
| RVA/Human-wt/PAK335/2015/G2P4 | MH236890 | RVA/Human-wt/PAK661/2015/G3P4 | MH109745 |
| RVA/Human-wt/PAK339/2015/G2P4 | MH109753 | RVA/Human-wt/PAK663/2015/G3P4 | MH109744 |
| **[P[6]** | | | |
| **Strain Name** | **Accession Number** | **Strain Name** | **Accession Number** |
| RVA/Human-wt/PAK1/2015/G9P6 | MH255681 | RVA/Human-wt/PAK209/2015/G12P6 | MH255676 |
| RVA/Human-wt/PAK3/2015/G9P6 | MH255682 | RVA/Human-wt/PAK222/2015/G12P6 | MH255694 |
| RVA/Human-wt/PAK4/2015/G9P6 | MH255683 | RVA/Human-wt/PAK224/2015/G12P6 | MH255695 |
| RVA/Human-wt/PAK7/2015/G9P6 | MH109777 | RVA/Human-wt/PAK230/2015/G12P6 | MH109791 |
| RVA/Human-wt/PAK9/2015/G9P6 | MH255688 | RVA/Human-wt/PAK257/2015/G12P6 | MH109792 |
| RVA/Human-wt/PAK13/2015/G9P6 | MH109778 | RVA/Human-wt/PAK261/2015/G12P6 | MH255696 |
| RVA/Human-wt/PAK25/2015/G12P6 | MH255693 | RVA/Human-wt/PAK307/2015/G12P6 | MH109793 |
| RVA/Human-wt/PAK34/2015/G9P6 | MH255684 | RVA/Human-wt/PAK328/2015/G12P6 | MH109794 |
| RVA/Human-wt/PAK41/2015/G1P6 | MH255685 | RVA/Human-wt/PAK333/2015/G12P6 | MH109795 |
| RVA/Human-wt/PAK46/2015/G9P6 | MH255687 | RVA/Human-wt/PAK343/2015/G12P6 | MH109796 |
| RVA/Human-wt/PAK60/2015/G12P6 | MH109779 | RVA/Human-wt/PAK348/2015/G12P6 | MH109797 |
| RVA/Human-wt/PAK62/2015/G1P6 | MH109780 | RVA/Human-wt/PAK361/2015/G12P6 | MH109798 |
| RVA/Human-wt/PAK71/2015/G9P6 | MH109781 | RVA/Human-wt/PAK380/2015/G12P6 | MH109799 |
| RVA/Human-wt/PAK75/2015/G12P6 | MH255690 | RVA/Human-wt/PAK440/2016/G1P6 | MH109800 |
| RVA/Human-wt/PAK79/2015/G12P6 | MH109782 | RVA/Human-wt/PAK441/2016/G12P6 | MH109801 |
| RVA/Human-wt/PAK81/2015/G12P6 | MH109783 | RVA/Human-wt/PAK442/2016/G12P6 | MH109802 |
| RVA/Human-wt/PAK86/2015/G1P6 | MH109784 | RVA/Human-wt/PAK446/2016/G12P6 | MH109803 |
| RVA/Human-wt/PAK92/2015/G12P6 | MH109785 | RVA/Human-wt/PAK447/2016/G12P6 | MH109804 |
| RVA/Human-wt/PAK96/2015/G1P6 | MH255679 | RVA/Human-wt/PAK467/2016/G12P6 | MH255692 |
| RVA/Human-wt/PAK102/2015/G9P6 | MH109786 | RVA/Human-wt/PAK490/2016/G9P6 | MH255689 |
| RVA/Human-wt/PAK127/2015/G3P6 | MH255691 | RVA/Human-wt/PAK493/2016/G9P6 | MH109805 |
| RVA/Human-wt/PAK197/2015/G1P6 | MH109787 | RVA/Human-wt/PAK500/2016/G9P6 | MH255680 |
| RVA/Human-wt/PAK202/2015/G9P6 | MH109788 | RVA/Human-wt/PAK592/2016/G12P6 | MH255686 |
| RVA/Human-wt/PAK203/2015/G9P6 | MH109789 | RVA/Human-wt/PAK598/2016/G12P6 | MH255677 |
| RVA/Human-wt/PAK207/2015/G9P6 | MH109790 | RVA/Human-wt/PAK635/2016/G12P6 | MH109806 |
| **P[8]** | | |  |
| RVA/Human-wt/PAK15/2015/G3P8 | MH109769 | RVA/Human-wt/PAK430/2016/G3P8 | MH109767 |
| RVA/Human-wt/PAK18/2015/G3P8 | MH109774 | RVA/Human-wt/PAK435/2016/G1P8 | MH109765 |
| RVA/Human-wt/PAK24/2015/G1P8 | MH255733 | RVA/Human-wt/PAK439/2016/G1P8 | MH255711 |
| RVA/Human-wt/PAK36/2015/G1P8 | MH255728 | RVA/Human-wt/PAK444/2016/G3P8 | MH109768 |
| RVA/Human-wt/PAK37/2015/G3P8 | MH255729 | RVA/Human-wt/PAK445/2016/G3P8 | MH109761 |
| RVA/Human-wt/PAK274/2015/G9P8 | MH255721 | RVA/Human-wt/PAK478/2016/G3P8 | MH109766 |
| RVA/Human-wt/PAK59/2015/G1P8 | MH109770 | RVA/Human-wt/PAK494/2016/G3P8 | MH109772 |
| RVA/Human-wt/PAK61/2015/G3P8 | MH255734 | RVA/Human-wt/PAK593/2016/G3P8 | MH255732 |
| RVA/Human-wt/PAK65/2015/G1P8 | MH255718 | RVA/Human-wt/PAK603/2016/G3P8 | MH109775 |
| RVA/Human-wt/PAK77/2015/G1P8 | MH255727 | RVA/Human-wt/PAK608/2016/G3P8 | MH255724 |
| RVA/Human-wt/PAK90/2015/G1P8 | MH255735 | RVA/Human-wt/PAK609/2016/G9P8 | MH255737 |
| RVA/Human-wt/PAK154/2015/G3P8 | MH109773 | RVA/Human-wt/PAK610/2016/G1P8 | MH255712 |
| RVA/Human-wt/PAK196/2015/G1P8 | MH255719 | RVA/Human-wt/PAK611/2016/G3P8 | MH255713 |
| RVA/Human-wt/PAK221/2015/G3P8 | MH255731 | RVA/Human-wt/PAK612/2016/G3P8 | MH255714 |
| RVA/Human-wt/PAK266/2015/G3P8 | MH255720 | RVA/Human-wt/PAK614/2016/G9P8 | MH255738 |
| RVA/Human-wt/PAK274/2015/G9P8 | MH255721 | RVA/Human-wt/PAK615/2016/G1G3P8 | MH255739 |
| RVA/Human-wt/PAK317/2015/G1P8 | MH255736 | RVA/Human-wt/PAK616/2016/G3P8 | MH109771 |
| RVA/Human-wt/PAK327/2015/G3P8 | MH109758 | RVA/Human-wt/PAK620/2016/G3P8 | MH255715 |
| RVA/Human-wt/PAK334/2015/G3P8 | MH255722 | RVA/Human-wt/PAK622/2016/G3P8 | MH255725 |
| RVA/Human-wt/PAK340/2015/G1P8 | MH109776 | RVA/Human-wt/PAK623/2016/G1P8 | MH255716 |
| RVA/Human-wt/PAK346/2015/G3P8 | MH109755 | RVA/Human-wt/PAK624/2016/G3P8 | MH255717 |
| RVA/Human-wt/PAK365/2015/G3P8 | MH255709 | RVA/Human-wt/PAK626/2016/G3P8 | MH255740 |
| RVA/Human-wt/PAK377/2015/G3P8 | MH109756 | RVA/Human-wt/PAK638/2016/G3P8 | MH109762 |
| RVA/Human-wt/PAK383/2015/G3P8 | MH255726 | RVA/Human-wt/PAK643/2016/G3P8 | MH109763 |
| RVA/Human-wt/PAK410/2015/G3P8 | MH109760 | RVA/Human-wt/PAK666/2016/G3P8 | MH109764 |
| RVA/Human-wt/PAK413/2015/G9P8 | MH109759 |  |  |
| **Strain Name** | **Accession Number** | **Strain Name** | **Accession Number** |
| **VP7 genotypes** | | | |
| **G1** | | | |
| RVA/Human-wt/PAK24/2015/G1P[8] | MH191269 | RVA/Human-wt/PAK231/2015/G1P[X] | MH062755 |
| RVA/Human-wt/PAK36/2015/G1P[8] | MH191270 | RVA/Human-wt/PAK317/2015/G1P[8] | MH191275 |
| RVA/Human-wt/PAK41/2015/G1P[6] | MH191271 | RVA/Human-wt/PAK340/2015/G1P[8] | MH191276 |
| RVA/Human-wt/PAK59/2015/G1P[8] | MH277407 | RVA/Human-wt/PAK435/2016/G1P[8] | MH191282 |
| RVA/Human-wt/PAK62/2015/G1P[6] | MH191281 | RVA/Human-wt/PAK439/2016/G1P[8] | MH062756 |
| RVA/Human-wt/PAK65/2015/G1P[8] | MH277408 | RVA/Human-wt/PAK440/2016/G1P[8] | MH062757 |
| RVA/Human-wt/PAK77/2015/G1P[8] | MH062758 | RVA/Human-wt/PAK540/2016/G1P[8] | MH191277 |
| RVA/Human-wt/PAK86/2015/G1P[8] | MH191272 | RVA/Human-wt/PAK601/2016/G1P[8] | MH191278 |
| RVA/Human-wt/PAK88/2015/G1P[8] | MH191273 | RVA/Human-wt/PAK610/2016/G1P[8] | MH191279 |
| RVA/Human-wt/PAK90/2015/G1P[8] | MH191274 | RVA/Human-wt/PAK623/2016/G1P[8] | MH191280 |
| **Strain Name** | **Accession Number** | **Strain Name** | **Accession Number** |
| **G2** | | | |
| RVA/Human-wt/PAK2/2015/G2P[4] | MH182472 | RVA/Human-wt/PAK205/2015/G2P[4] | MH182474 |
| RVA/Human-wt/PAK6/2015/G2P[4] | MH109864 | RVA/Human-wt/PAK268/2015/G2P[4] | MH109860 |
| RVA/Human-wt/PAK12/2015/G2P4 | MH277404 | RVA/Human-wt/PAK335/2015/G2P[4] | MH109861 |
| RVA/Human-wt/PAK43/2015/G2P[4] | MH109865 | RVA/Human-wt/PAK347/2015/G2P4 | MH277406 |
| RVA/Human-wt/PAK49/2015/G2P[4] | MH182473 | RVA/Human-wt/PAK350/2015/G2P[4] | MH182475 |
| RVA/Human-wt/PAK70/2015/G2P[4] | MH109866 | RVA/Human-wt/PAK577/2016/G2P[4] | MH109862 |
| RVA/Human-wt/PAK147/2015/G2P[4] | MH109868 | RVA/Human-wt/PAK653/2016/G2P[4] | MH109863 |
| RVA/Human-wt/PAK167/2015/G2P[4] | MH109867 |  |  |
| **Strain Name** | **Accession Number** | **Strain Name** | **Accession Number** |
| **G3** | | | |
| RVA/Human-wt/PAK15/2015/G3P8 | MH279579 | RVA/Human-wt/PAK502/2016/G3P4 | MH279584 |
| RVA/Human-wt/PAK18/2015/G3P8 | MH279586 | RVA/Human-wt/PAK430/2016/G3P8 | MH109813 |
| RVA/Human-wt/PAK37/2015/G3P8 | MH279578 | RVA/Human-wt/PAK445/2016/G3P8 | MH109822 |
| RVA/Human-wt/PAK38/2015/G3P4 | MH279569 | RVA/Human-wt/PAK478/2016/G3P8 | MH109823 |
| RVA/Human-wt/PAK60B/2015/G3P8 | MH109807 | RVA/Human-wt/PAK558/2016/G3P8 | MH279593 |
| RVA/Human-wt/PAK78/2015/G3P8 | MH109808 | RVA/Human-wt/PAK593/2016/G3P8 | MH279592 |
| RVA/Human-wt/PAK127/2015/G3P6 | MH279585 | RVA/Human-wt/PAK603/2016/G3P8 | MH279571 |
| RVA/Human-wt/PAK154/2016/G3P8 | MH279577 | RVA/Human-wt/PAK604/2016/G3P4 | MH109814 |
| RVA/Human-wt/PAK266/2015/G3P8 | MH109809 | RVA/Human-wt/PAK608/2016/G3P8 | MH109824 |
| RVA/Human-wt/PAK298/2015/G3P4 | MH279570 | RVA/Human-wt/PAK611/2016/G3P8 | MH279573 |
| RVA/Human-wt/PAK316/2015/G3P4 | MH109810 | RVA/Human-wt/PAK612/2016/G3P8 | MH109825 |
| RVA/Human-wt/PAK327/2015/G3P8 | MH109819 | RVA/Human-wt/PAK615/2016/G3P8 | MH279591 |
| RVA/Human-wt/PAK334/2015/G3P8 | MH109820 | RVA/Human-wt/PAK616/2016/G3P8 | MH279572 |
| RVA/Human-wt/PAK339/2015/G3P4 | MH279582 | RVA/Human-wt/PAK620/2016/G3P8 | MH109826 |
| RVA/Human-wt/PAK346/2015/G3P8 | MH109821 | RVA/Human-wt/PAK624/2016/G3P8 | MH109815 |
| RVA/Human-wt/PAK365/2015/G3P8 | MH109811 | RVA/Human-wt/PAK626/2016/G3P8 | MH279574 |
| RVA/Human-wt/PAK377/2015/G3P8 | MH279587 | RVA/Human-wt/PAK638/2016/G3P8 | MH279576 |
| RVA/Human-wt/PAK382/2015/G3P4 | MH279581 | RVA/Human-wt/PAK643/2016/G3P8 | MH109816 |
| RVA/Human-wt/PAK383/2015/G3P8 | MH279588 | RVA/Human-wt/PAK650/2016/G3P4 | MH109817 |
| RVA/Human-wt/PAK410/2016/G3P8 | MH279589 | RVA/Human-wt/PAK661/2016/G3P4 | MH279580 |
| RVA/Human-wt/PAK419/2016/G3P4 | MH109812 | RVA/Human-wt/PAK663/2016/G3P4 | MH109818 |
| RVA/Human-wt/PAK425/2016/G3P8 | MH279590 | RVA/Human-wt/PAK666/2016/G3P8 | MH279575 |
| **Strain Name** | **Accession Number** | **Strain Name** | **Accession Number** |
| **G9** | | | |
| RVA/Human-wt/PAK1/2015/G9P6 | MH109853 | RVA/Human-wt/PAK171/2015/G9P6 | MH109849 |
| RVA/Human-wt/PAK3/2015/G9P6 | MH109854 | RVA/Human-wt/PAK202/2015/G9P8 | MH277395 |
| RVA/Human-wt/PAK4/2015/G9P6 | MH109858 | RVA/Human-wt/PAK203/2015/G9P6 | MH109869 |
| RVA/Human-wt/PAK7/2015/G9P6 | MH109847 | RVA/Human-wt/PAK274/2015/G9P8 | MH109850 |
| RVA/Human-wt/PAK9/2015/G9P6 | MH109848 | RVA/Human-wt/PAK411/2015/G9PX | MH277398 |
| RVA/Human-wt/PAK13/2015/G9P6 | MH109855 | RVA/Human-wt/PAK413/2016/G9P8 | MH277400 |
| RVA/Human-wt/PAK34/2015/G9P6 | MH277394 | RVA/Human-wt/PAK490/2016/G9P6 | MH109851 |
| RVA/Human-wt/PAK46/2015/G9P6 | MH277402 | RVA/Human-wt/PAK493/2016/G9P6 | MH109852 |
| RVA/Human-wt/PAK56/2015/G9P8 | MH109856 | RVA/Human-wt/PAK500/2016/G9P6 | MH109859 |
| RVA/Human-wt/PAK71/2015/G9P6 | MH109857 | RVA/Human-wt/PAK516/2016/G9PX | MH277397 |
| RVA/Human-wt/PAK102/2015/G9P6 | MH277401 | RVA/Human-wt/PAK609/2016/G9P8 | MH277399 |
| RVA/Human-wt/PAK166/2015/G9P6 | MH277396 | RVA/Human-wt/PAK614/2016/G9P8 | MH109870 |
| **Strain Name** | **Accession Number** | **Strain Name** | **Accession Number** |
| **G12** | | | |
| RVA/Human-wt/PAK25/2015/G12P6 | MH255697 | RVA/Human-wt/PAK348/2015/G12P6 | MH255703 |
| RVA/Human-wt/PAK60A/2015/G12P6 | MH255698 | RVA/Human-wt/PAK343/2015/G12P6 | MH109839 |
| RVA/Human-wt/PAK75/2015/G12P6 | MH255699 | RVA/Human-wt/PAK361/2015/G12P6 | MH109840 |
| RVA/Human-wt/PAK79/2015/G12P6 | MH109827 | RVA/Human-wt/PAK367/2015/G12P6 | MH109841 |
| RVA/Human-wt/PAK81/2015/G12P6 | MH109828 | RVA/Human-wt/PAK380/2015/G12P6 | MH255704 |
| RVA/Human-wt/PAK92/2015/G12P6 | MH255700 | RVA/Human-wt/PAK402/2015/G12P6 | MH255705 |
| RVA/Human-wt/PAK207/2015/G12P6 | MH255701 | RVA/Human-wt/PAK441/2016/G12P6 | MH109842 |
| RVA/Human-wt/PAK209/2015/G12P6 | MH109836 | RVA/Human-wt/PAK442/2015/G12P6 | MH255706 |
| RVA/Human-wt/PAK222/2015/G12P6 | MH255702 | RVA/Human-wt/PAK446/2016/G12P6 | MH109833 |
| RVA/Human-wt/PAK230/2015/G12P6 | MH109837 | RVA/Human-wt/PAK447/2016/G12P6 | MH109834 |
| RVA/Human-wt/PAK257/2015/G12P6 | MH109829 | RVA/Human-wt/PAK467/2016/G12P6 | MH109843 |
| RVA/Human-wt/PAK261/2015/G12P6 | MH109830 | RVA/Human-wt/PAK547/2016/G12P6 | MH109844 |
| RVA/Human-wt/PAK307/2015/G12P6 | MH109838 | RVA/Human-wt/PAK592/2016/G12P6 | MH255707 |
| RVA/Human-wt/PAK328/2015/G12P6 | MH109831 | RVA/Human-wt/PAK598/2015/G12P6 | MH255708 |
| RVA/Human-wt/PAK333/2015/G12P6 | MH109832 | RVA/Human-wt/PAK635/2016/G12P6 | MH109835 |
