# Supplementary figures and images for: Molecular characterization of human group A rotavirus genotypes circulating in Rawalpindi, Islamabad, Pakistan during 2015-2016
Source: PLoS One. 2019 Jul 30;14(7):e0220387. doi: 10.1371/journal.pone.0220387 (PMC6667158; doi:10.1371/journal.pone.0220387)

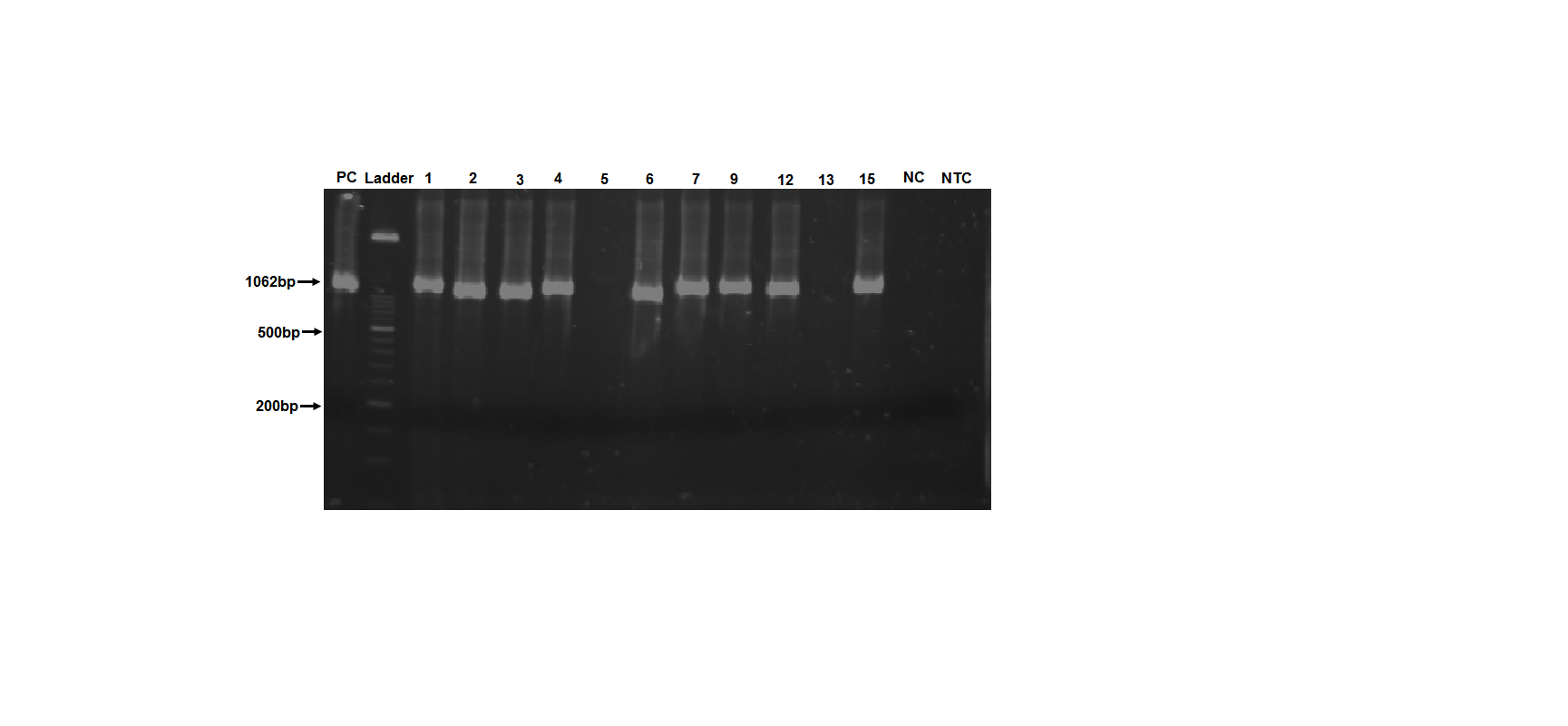

Supplement: S1 Fig — Lane 1: positive control, Lane 2: DNA ladder 50 bp, lane 3, 4, 5, 6, 8, 9, 10, 11, 13: Amplified product of gene segment VP7(1062 bp size) of samples no 1, 2, 3, 4, 6, 7, 9, 12 and 15, Lane7, 12: Samples number 5, 13 negative for VP7 gene, Lane 14, 15: Negative control and non-target control(NT-C), respectively. (TIF) [file pone.0220387.s002.tif]

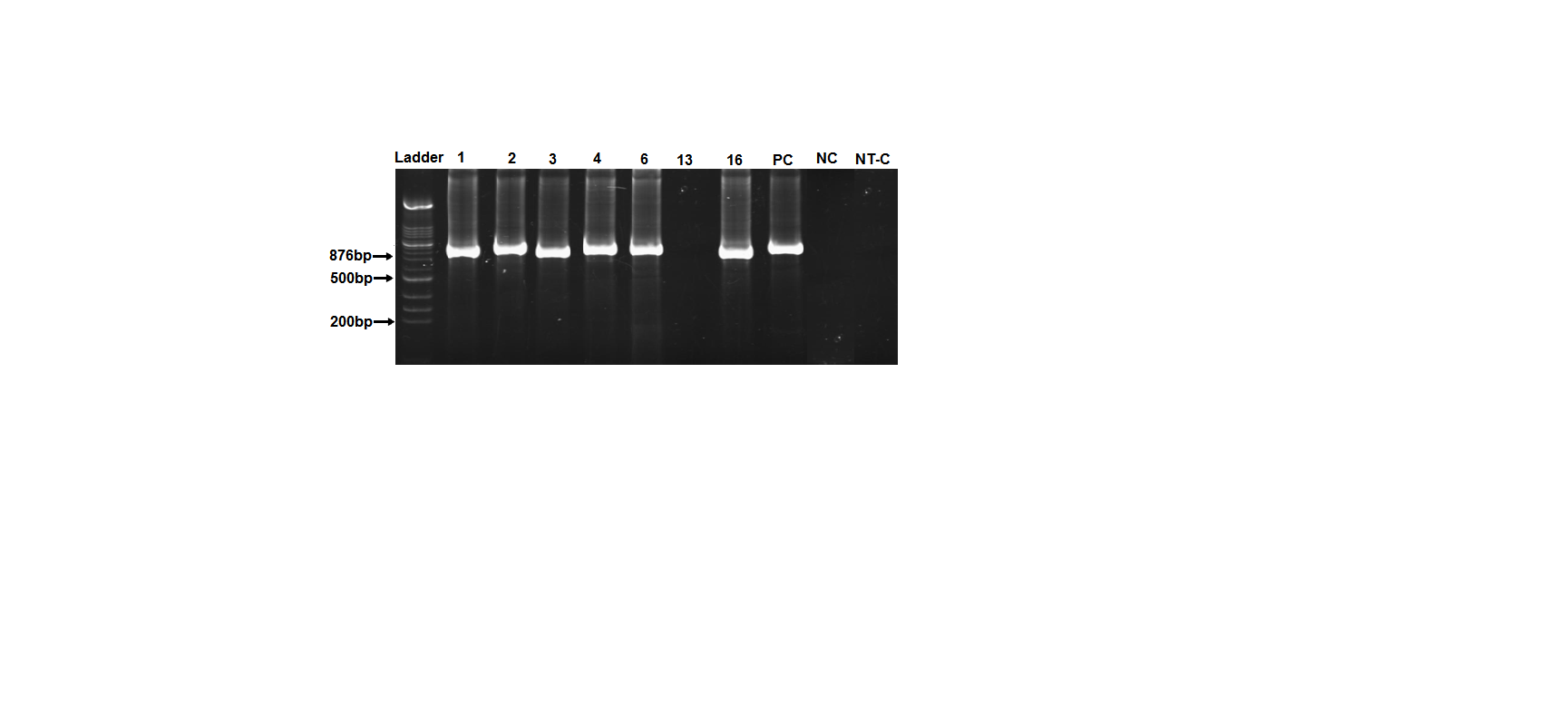

Supplement: S2 Fig — Lane 1: DNA ladder 50 bp, lane 2, 3, 4, 5, 6, 8: Amplified product of gene segment VP4 (876 bp size) of samples no 1, 2, 3, 4, 6 and 16, Lane7: Samples number 13 negative for VP4 gene, Lane 9, 10 and 11: Positive control, Negative control and non-target control (NT-C), respectively. (TIF) [file pone.0220387.s003.tif]
